# Encrypted Antimicrobial Peptides from Plant Proteins

**DOI:** 10.1038/s41598-017-13685-6

**Published:** 2017-10-16

**Authors:** M. H. S. Ramada, G. D. Brand, F. Y. Abrão, M. Oliveira, J. L. Cardozo Filho, R. Galbieri, K. P. Gramacho, M. V. Prates, C. Bloch

**Affiliations:** 1 Laboratório de Espectrometria de Massa, Embrapa Recursos Genéticos e Biotecnologia, 70770-917 Brasília, DF Brazil; 2 Departamento de Biologia Celular, Instituto de Ciências Biológicas, Universidade de Brasília, 70910-900 Brasília, DF Brazil; 3 Laboratório de Síntese e Análise de Biomoléculas, Instituto de Química, Universidade de Brasília, Brasília, DF Brazil; 4 Faculdade de Farmácia, FacUnicamps, 74535-280 Goiânia, GO Brazil; 5 Departamento de Fitopatologia, Instituto Mato-Grossense do Algodão, 78850-000 Primavera do Leste, MT Brazil; 6 Laboratório de Fitopatologia Molecular, Centro de Pesquisa do Cacau, 45600-970 Itabuna, BA Brazil; 7 Present Address: Pós-Graduação em Ciências Genômicas e Biotecnologia, Universidade Católica de Brasília, 70790-160 Brasília, DF Brazil

## Abstract

Examples of bioactive peptides derived from internal sequences of proteins are known for decades. The great majority of these findings appear to be fortuitous rather than the result of a deliberate and methodological-based enterprise. In the present work, we describe the identification and the biological activities of novel antimicrobial peptides unveiled as internal fragments of various plant proteins founded on our hypothesis-driven search strategy. All putative encrypted antimicrobial peptides were selected based upon their physicochemical properties that were iteratively selected by an in-house computer program named Kamal. The selected peptides were chemically synthesized and evaluated for their interaction with model membranes. Sixteen of these peptides showed antimicrobial activity against human and/or plant pathogens, some with a wide spectrum of activity presenting similar or superior inhibition efficacy when compared to classical antimicrobial peptides (AMPs). These original and previously unforeseen molecules constitute a broader and undisputable set of evidences produced by our group that illustrate how the intragenic concept is a workable reality and should be carefully explored not only for microbicidal agents but also for many other biological functions.

## Introduction

In Greek Philosophy but also under the historical knowledge accumulated in Physics, Aristotle introduced the concept of *first cause* or *prime mover*, meaning the first of all substances or the commencement of everything that exists in nature. Assuming that complex and superior beings are not logically supposed to be the unique cause (origin, explanation) of themselves, under the framework proposed by the thinker, things in the universe should be caused by previous and original ones. Therefore, these causes exist in succession, as a chain of events distending regressively.

The paradigmatic cosmological theory of the Big Bang, initially suggested in Georges Lemaître’s pivotal work, “*Un univers homogène de masse constante et de rayon croissant rendant compte de la vitesse radiale des nébuleuses extra-galactiques*”^1^ and his subsequent Primeval-Atom hypothesis^2,3^; the Universal Common Ancestry theory^4^, strengthened by the recent advances in genome sequencing; or the present models of development and differentiation of stem cells^5,6^, seem to share the same archetypical structure as proposed by the Aristotelian framework. It means that all these theories and models conceive a fundamental starting point of its own substance that during a certain period of time moves towards an increasing structural and functional complexity until the inevitable finitude. By analogy, the complete genome of any living form could also be considered as the primordial starting point in a chain of events. It means that a linearly organized genetic material undergoes metabolic processing yielding structures with forms and functions that, in many ways, as a direct function of time and environmental conditions, appear to be different or substantially apart from its origin. Considering a broader and interspecific scenario, the actual differences in shapes, sizes and behavior our senses perceive in all living creatures, may also be seen as successful products of the internal possibilities (potentialities) of the prime genetic material exposed to a number of evolutionary factors in the time line.

In our previous work^7^, inspired by this theoretical reasoning, protein sequences were perceived as the prime source from where antimicrobial peptides (AMPs) could be located in one or more regions of the complete polypeptide chain of some mature and expressed proteins. In strict tandem with Aristotle’s thought, the primary structures of certain proteins were assumed to be the *material cause* of our system, i.e. the raw material (*potentiality*) from which antimicrobial peptides (*actuality*) could be physically attained in the same manner that in the cytoplasm newly translated precursor polypeptides (pre-pro-proteins) from correspondent mRNA’s are enzymatically processed into mature proteins and/or peptides. The practical differences of our approach rest on what Aristotle’s analytical method defined as *formal*, *final and efficient causes*. In short, an in-house made computer program, called Kamal, generates interactive routines of *in silico* processing of protein sequences oriented by specific physicochemical parameters capable of identifying putative antimicrobial peptide fragments inside larger polypeptide chains^7^. These physicochemical parameters, e.g. net charge, average hydrophobicity, and hydrophobic moment, were originally derived from native AMPs described in the literature and used as archetypal parameters to define antimicrobial activity, or in Aristotelian terms, the *formal cause*. Protein fragments with matching physicochemical properties to the above-described antimicrobial archetype were filtered, chemically synthesized, and tested accordingly to evaluate their microbicidal function (*final cause*). Evidences of “encrypted” protein fragments showing dissimilar biological activities to their original parent-molecules have been available for some time^8–13^. However, to our best knowledge, a systematic hypothesis-driven initiative to produce analytical means able to rationally decipher unseen bioactive potentialities inside protein primary structures into biologically relevant functional actualities in the form of microbicidal molecules or “intragenic” antimicrobial peptides (IAPs) was first demonstrated by our group^7,14,15^.

Our methodology relies in two reinforcing steps: the filtering of genomes/protein collections using Kamal, and a classification tool to identify the IAPs that induce comparable disturbances in model phospholipid membranes. The physicochemical properties of peptide subgroups associated with potent antimicrobial activity are fed back to Kamal and used for successive rounds of peptide filtering/evaluation^7^. In fact, our system mimics an evolutionary process, with the generation of diversity followed by the selection of the fittest. The present work reports a substantial quantitative and qualitative improvement of our methodology on bioactive peptide screening by showing a new generation of IAPs with antibacterial and antifungal activities obtained from various protein sequences belonging to different plants. The biological activities of two frog skin antimicrobial peptides, DS01 from *Phyllomedusa oreades* and Ascaphin-8 from *Ascaphus truei*, are also evaluated as references of native AMPs^16,17^. Our work demonstrates that intragenic antimicrobial peptides can be as active as known AMPs, with similar selectivity and breadth of antimicrobial spectrum. Furthermore, it provides additional evidence that organisms expressing intragenic peptides could be a feasible alternative to transgenic technology using conventional AMPs^18,19^ in the constant need for novel agents for the control of pathogens.

## Results

### Plants genomes as sources of IAPs

**Figure 1:**
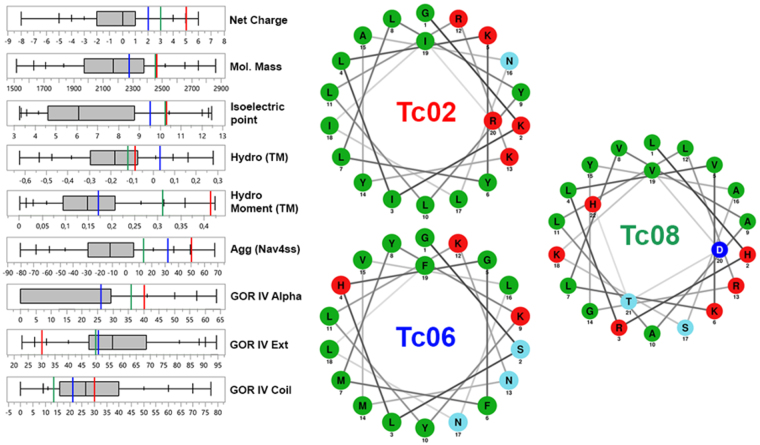
Physicochemical parameters and helical wheel plot of the IAPs Tc02, Tc06 and Tc08. Quantile box plots of the net charge, molecular mass (Da), isoelectric point^21^, average hydrophobicity (TM scale^23^), Hydrophobic moment (TM scale^23^), Aggregation (Nav4SS parameter from Aggrescan^22^), GOR IV^53^ alpha helix, extended configuration and coil structures was generated for 10.000 randomly selected peptides ranging from 16–22 amino acids from *T*. *cacao* genome. Color bars in each quantile plot represent the physicochemical parameters for the IAPs Tc02 (red), Tc06 (blue) and Tc08 (green). Helical wheel plots for the same IAPs are also represented, demonstrating that once structured as α-helical segments, these peptides show an amphiphilic character similar to classical AMPs. Hydrophobic residues are represented in green, positively charged residues are represented in red, negatively charged residues are represented in blue and polar uncharged residues in light blue. Tc02, Tc06 and Tc08 were selected based on their tested antimicrobial activities (Tables 4 and 5) to illustrate physicochemical parameters of the filtered IAPs when compared to randomly selected peptides from *T*. *cacao*. Physicochemical properties and helical wheel plots of the remaining IAPs are shown on Supplementary Material 01.

Publicly available protein collections from *Theobroma* cacao, *Arabidopsis thaliana*, *Citrus sinensis* and *Gossypium*. *raimondii* were submitted to the software Kamal v1.0 *alpha* for the filtering of putative IAPs encrypted within proteins. Kamal uses calculated physicochemical properties derived from protein primary structure as descriptors of peptide activity, as previously demonstrated by our group^7^. Nine filters were sequentially applied to plant genomes to uncover putative IAPs, as described in Table 1. Kamal generates a list of protein fragments with higher probability of antimicrobial activity, which should be curated by the user aided by auxiliary tools available online^20^. By extensive user examination of the putative IAPs generated by Kamal, twenty-one peptides were selected for solid phase peptide synthesis (Table 2). Tc02, Tc06 and Tc08 have their helical wheel projections and their physicochemical properties depicted in Fig. 1 as an illustration. The distribution of amino acid residues in these IAPs in theoretical helical segments emulates the characteristic amphiphilic character of AMPs^20^. Furthermore, to investigate the physicochemical properties of putative IAPs prospected from the *T*. *cacao* genome in relation to randomly selected peptides, quantile plots were generated. Tc02, Tc06 and Tc08 have a net charge, isoelectric point^21^ and an aggregation tendency (Na4vSS)^22^ higher than the 3^rd^ quartile of a sample of ten thousand peptides from 16 to 22 amino acid residues prospected randomly from the same *T*. *cacao* protein collection. Moreover, average hydrophobicity^23^ and hydrophobic moment^24^ are in the third quartile of the reference set (Fig. 1). These three peptides are representative of the putative IAPs derived from plant genomes selected for solid phase peptide synthesis (Supplementary Material 01, Table S1 and Figure S1). Therefore, putative IAPs selected for synthesis tend to be more cationic, more hydrophobic, and more prone to aggregation than the average *T*. *cacao* random protein fragment of the same number of residues.

**Table 1:** User-defined physicochemical properties used for the search of IAPs.

Physicochemical Parameters	Window Range	
Peptide size	16–22 aa	
Residue filter	C and P*	
Net Charge	+1 up to +5	
Molecular Mass (Da)	1400 up to 4000	
Hydrophobicity Tm scale^23^	−0.485 up to +0.516	
Hydrophobic Moment Tm scale^23^	+0.516 up to +1.267	
Aggregation^22^	−10 up to +55	
Isoelectric Point^21^	9 up to 13	
Windowed Hydrophobicity^54^	Max 1 up to 3	Min −2 up to −0.5

*Exclude peptides with cysteine (C) or proline (P) residues.

**Table 2:** Peptide IDs, source proteins, scanned organisms, peptide sequences and number of residues of the current generation of IAPs.

Peptide ID	Source protein	Organism (s)	Peptide Sequence (*)	N.r.
Tc01	Embryo defective 1381 isoform 2	*Theobroma cacao*	**VALRLAKEVIKVQQGW**	16
Tc02	ARM repeat superfamily protein, putative isoform 1	*Theobroma cacao*	**GKILKYLLYLLRKYANLIIR**	20
Tc03	Tc05_g014340 (12–32)**	*Theobroma cacao*	**IKLRNVLKYLFRIDVIKEDIL**	21
Tc04	Purple acid phosphatase 3	*Theobroma cacao*	**RVLKDVESALRESVANWKIVIG**	22
Tc05	Gamma interferon responsive lysosomal thiol reductase family protein, putative	*Theobroma cacao*	**IVNHLVKLFDKGLNSIVNLR**	20
Tc06	Cytochrome P450, family 87, subfamily A, polypeptide 2	*Theobroma cacao*	**GSLHGFMYKYLKNMVLNLF**	19
Tc07	Laccase 17	*Theobroma cacao*	**LIKVVNHVQYNVTLHWHGIR**	20
Tc08	Uncharacterized protein TCM_026695	*Theobroma cacao*	**LHRLVKLVAALLRGYASKVDTH**	22
Tc09	NB-ARC domain-containing disease resistance protein, putative	*Theobroma cacao*	**GIVLKDLFSEKLRRYKIVIG**	20
Tc10	NB-ARC domain-containing disease resistance protein, putative	*Theobroma cacao*	**GLLFKELQKLIRYQIFIGK**	19
Tc11	LRR and NB-ARC domains-containing disease resistance protein, putative isoform 1	*Theobroma cacao*	**LLDKLKRTLLSIEAVLI**	17
At01	Cytochrome P450, family 87, subfamily A, polypeptide 2	*Arabidopsis thaliana*	**GSLHGFMYKYLKNMVLTLF**	19
At02	SIP121-0-PQ4 (45-60)***	*Arabidopsis thaliana*	**KVLSKVHTLLKAVLAL**	16
At03	YELLOW STRIPE like	*Arabidopsis thaliana*	**GAKLAKKQVRALGKFFSF**	18
At04	YELLOW STRIPE like	*Arabidopsis thaliana*	**GLYNFIKVLGRTVFGLYKQF**	20
Cs01	PREDICTED: cytochrome P450 87A3	*Citrus sinensis*	**GSLHGFMYRYLKNMVLNLF**	19
Zm01	PREDICTED: cytochrome P450 87A3-like	*Zea mays*	**GSLHGFMYKYLKTLVLRLY**	19
Cs02	photosystem II CP47 chlorophyll apoprotein (chloroplast)	*Citrus sinensis*	**FFGHIWHGARTLFRDVFA**	18
Cs03	hypothetical protein CISIN_1g015590mg	*Citrus sinensis*	**FFYNVIKIYGNMAGRISK**	18
Gr01	PREDICTED: uncharacterized protein LOC105765114	*Gossypium raimondii*	**GFKLGRKLVKVFKWII**	16
Gr02	hypothetical protein B456_004G254100	*Gossypium raimondii*	**ANRLLEAYKMLLKFLGNLR**	19

^*^Carboxyamidated peptides. ^**^Protein not available on NCBInr database. This protein was obtained from the predicted proteins downloaded from cocoagendb.cirad.fr. ^***^Protein nor available on NCBInr database. This protein was obtained from a list of oxidative-stress from *A*. *thaliana* ^55^.

### The interaction of IAPs with model membranes

**Figure 2:**
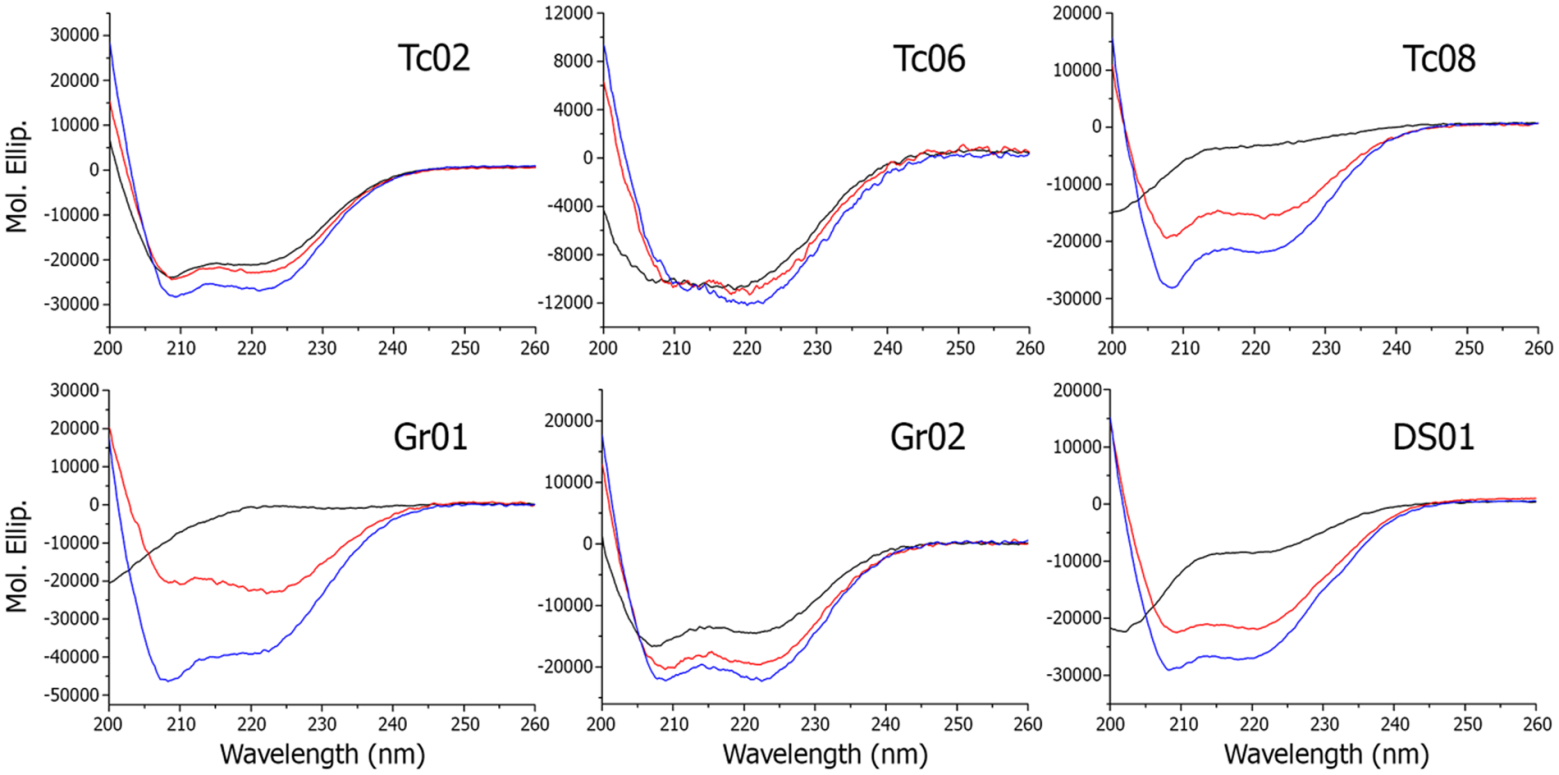
Circular Dichroism (CD) spectra of IAPs. Far-UV CD scans were performed to evaluate the secondary structure of IAPs in buffer alone (40 μM solution peptide in phosphate buffer), represented as a black line, and after the addition of 2 mM DMPC, represented as a red line, or in the presence of 2 mM 2:1 DMPC:DMPG, blue line. Tc02 and Gr02 dichroic bands indicate that both peptides are already partially structured as α-helical segments in buffer, and the addition of LUVs induces further structuration. Tc08, Gr01 and DS01 are randomly structured in buffer, only showing α-helix dichroic bands in the presence of LUVs.

The secondary structure of synthetic IAPs was investigated in buffer and after the addition of dimyristoilphophatidilcoline (DMPC) and 2:1 dimyristoilphophatidilcoline: dimyristoilphophatidilglycerol (DMPC:DMPG) large unilamelar vesicules (LUVs) at 50-fold molar excess. In buffer, most peptides presented far Ultraviolet-Circular Dichroism (UV-CD) spectra compatible with random coil structures (Table 3). Nevertheless, some were already structured as α-helices or β-sheets even before the addition of vesicles. Peptides Tc02 and Gr02 are structured as stable α-helical segments in buffer, and membrane addition accentuated their helical content (Fig. 2). IAPs, such as At02, Gr01 and Tc08, transitioned from random coil structures in buffer to α-helical segments upon membrane interaction, similarly to the classical AMP DS01^7,16,25^ (Fig. 2). Other molecules presented Far-UV CD spectra compatible with β-sheets and were considered separately. The helical percentages of the 21 IAPs evaluated herein in buffer and following the addition of LUVs are described in Table 3. This data corroborates our previous results that IAPs behaves as most membrane-active α-helical AMPs undergoing structure change in the presence of membranes.

**Table 3:** Helical percentages of IAPs with or without LUVs.

Peptide ID	%Hel buffer	%Hel 2:1 DMPC/DMPG	%Hel DMPC
Tc01	5.0	64.8	4.5
Tc02	60.4	77.0	65.6
Tc03	*	50.7	36.8
Tc04	14.3	36.3	15.8
Tc05	8.3	68.8	34.9
Tc06	29.4	34.9	31.5
Tc07	23.5	19.9	17.1
Tc08	9.0	62.1	40.4
Tc09	12.3	0.0	19.3
Tc10	20.3	49.6	5.1
Tc11	45.8	56.7	57.0
At01	32.4	28.4	26.4
At02	13.3	79.8	32.6
At03	0.0	30.8	0.0
At04	*	*	*
Cs01	26.3	27.5	37.8
Zm01	31.0	65.5	42.2
Cs02	4.3	52.9	52.8
Cs03	*	*	*
Gr01	0.9	115.0	57.7
Gr02	56.7	64.2	50.5
Asc-8	2.7	88.5	74.2
DS01	23.2	72.4	59.4

*β-sheet structure.

**Figure 3:**
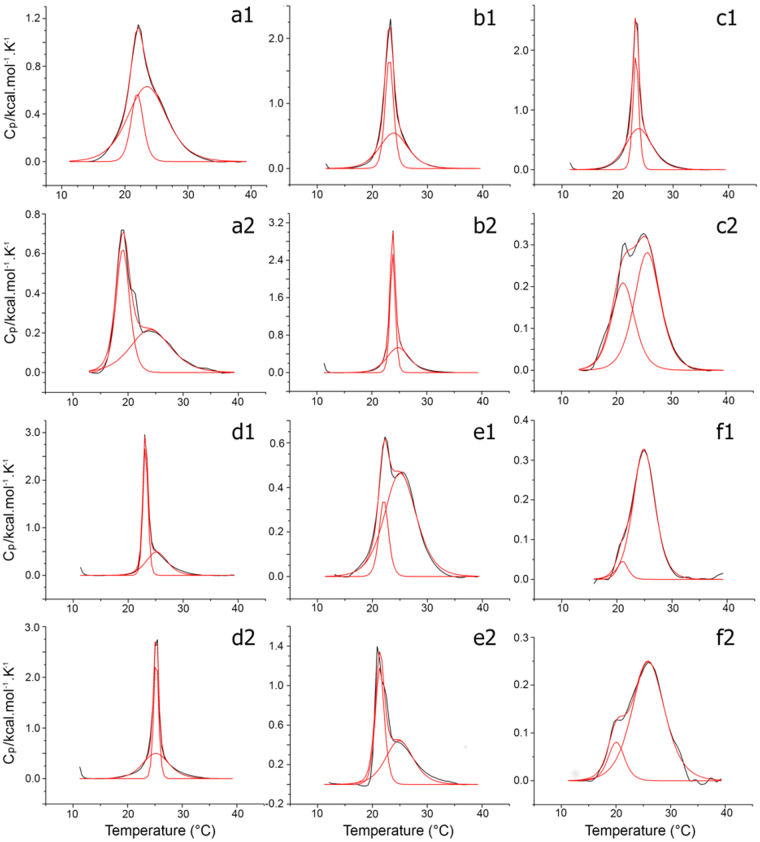
Heating thermal scans of DMPC and 2:1 DMPC:DMPG LUVs enriched with 4 mol% IAPs. Non two-state model fitting of P′_β_ → L_α_ phase transition of a solution of 0.5 mM of (1) DMPC or (2) 2:1 DMPC:DMPG LUVs enriched with 4 mol% of Tc02 (**a**), Tc06 (**b**), Tc08 (**c**), Gr01 (**d**), Gr02 (**e**) or DS01 (**f**). Black line corresponds to experimental data. Red line corresponds to model fitting.

We have further investigated the thermotropic behavior of phospholipid membranes enriched with 4 mol% IAPs by differential scanning calorimetry (Fig. 3). The putative IAPs varied in their ability to alter the P′_β_→L_α_ main phase transition of DMPC and 2:1 DMPC:DMPG LUVs. Peptides Tc02 and Gr02 disturbed the main phase transition of vesicles similarly to DS01 (Fig. 3). These two IAPs induced a two-component main phase transition with a more cooperative endotherm at a lower temperature (sharp component) superimposed by another less-cooperative endotherm at a higher temperature (broad component). These alterations are characteristic of deep peptide penetration in the acyl interior of model membranes, a feature previously associated with antimicrobial activity^7^. Other peptides, such as Gr01, disturbed the main phase transition of LUVs to a lesser extent, indicating that although this peptide structures as an α-helical segment, it interacts more superficially with model membranes (Fig. 3).

### IAPs are potent and broad antimicrobial agents with low hemolytic activities

The antimicrobial activity of the 21 putative IAPs versus Gram-positive and Gram-negative bacteria, yeasts and filamentous fungi were evaluated. A panel of plant and human pathogenic microorganisms were assayed. Sixteen IAPs, or approximately 76% of the synthesized IAPs, showed inhibitory activity at concentrations up to 256 µM against at least one of the evaluated microorganisms (Tables 4 and 5). Five of them, Tc02, Tc08, At02, Gr01 and Gr02 were as potent as DS01^16^ and Asc-8^17^, with a similar amplitude of spectrum of activity. Moreover, some specificity towards particular groups of microorganisms were observed. Tc02 inhibited the growth of yeasts and filamentous fungi more efficiently than Gram-positive and -negative bacteria, while Tc08 presented an opposite pattern of activity. The same is true for At04, which showed almost negligible activity to most microorganisms, except for *Staphylococcus aureus*. Tc06, At01, Cs01 and Zm01 were also as potent as AMPs in inhibiting the growth of yeasts. Other peptides, such as At02, Gr01 and Gr02, inhibited the growth of microorganisms without regard to specific membrane structures or phospholipid compositions.

**Table 4:** Antimicrobial activity of IAPs against yeasts and filamentous fungi.

Peptides	Minimum inhibitory concentration (µM ± SD)*							
	*Candida albicans*	*Cryptococcus neoformans*	*Fusarium solani*	*Fusarium oxysporum*	*Fusarium oxysporum f*.*sp*. *vasinfectum*	*Ramularia areola*	*Rhizoctonia solani*	*Trichoderma asperellum*
Tc02	1.0	1.0	1.0	2.0	0.83 ± 0.29	0.83 ± 0.29	1.0	1.0
Tc03	N/A	N/A	N/A	N/A	N/A	N/A	N/A	N/A
Tc05	128.0	128.0	N/A	N/A	N/A	N/A	N/A	N/A
Tc06	4.0	10.67 ± 4.61	32.0	16.0	16.0	4.0	N/A	6.67 ± 2.31
Tc08	4.0	8.0	32.0	32.0	2.0	4.0	16.0	16.0
Tc10	64.0	64.0	N/A	N/A	N/A	64.0	N/A	N/A
At01	16.0 ± 0.0	16.0 ± 0.0	32.0 ± 0.0	16.0 ± 0.0	8.0 ± 0.0	4.0 ± 0.0	N/A	8.0 ± 0.0
At02	8.0 ± 0.0	4.0 ± 0.0	16.0 ± 0.0	4.0 ± 0.0	4.0 ± 0.0	2.0 ± 0.0	16.0 ± 0.0	16.0 ± 0.0
At03	128.0 ± 0.0	64.0 ± 0.0	N/A	256.0 ± 0.0	64.0 ± 0.0	16.0 ± 0.0	N/A	128.0 ± 0.0
At04	N/A	64,0 ± 0,0	N/A	N/A	N/A	N/A	N/A	N/A
Cs01	2.67 ± 1.15	8.0	16.0	32.0	8.0	4.0	N/A	32.0
Zm01	6.67 ± 2.31	4.0	4.0	16.0	10.67 ± 4.61	2.0	32.0	16.0
Cs02	32.0	32.0	8.0	16.0	13.33 ± 4.61	16.0	32.0	8.0
Cs03	128.0	32.0	128.0	128.0	128.0	64.0	N/A	256.0
Gr01	1.0	1.0	0.5	0.5	0.5	0.5	1.0	1.0
Gr02	4.0	2.67 ± 1.15	2.0	4.0	1.67 ± 0.57	2.0	4.0	4.0
Asc-8	8.0	4.0	4.0	8.0	2.0	4.0	16.0	3.33 ± 1.15
DS01	4.0	8.0	16.0	16.0	4.0	8.0	64.0	8.0
Fluconazole	1.63	1.63	N/A	N/A	26.12	N/A	N/A	N/A
Amphotericin B	0.27	0.13	1.08	0.54	1.08	0.54	1.08	2.16

^*^Standard deviation equals 0 is not displayed in the table. N/A – No antimicrobial activity observed. Peptides Tc01, Tc04, Tc07, Tc09 and Tc11 did not show antimicrobial activity against any of the tested microorganisms.

**Table 5:** Antimicrobial activity of IAPs against Gram–positive and –negative bacteria.

Peptides	Minimum inhibitory concentration (µM ± SD)							
	*Escherichia coli*	*Pseudomonas aeruginosa*	*Bacillus subtilis*	*Bacillus cereus*	*Staphylococcus aureus*	*Erwinia carotovora*	*Pseudomonas syringae pv*. *tabaci*	*Xanthomonas campestris*
Tc02	10.67 ± 4.61	16.0	4.0	13.33 ± 4.61	8.0	16.0	4.0	16.0
Tc03	64.0	N/A	16.0	128.0	N/A	32.0	32.0	64.0
Tc05	N/A	N/A	64.0	N/A	N/A	N/A	N/A	N/A
Tc06	32.0	26.67 ± 9.24	16.0	64.0	13.33 ± 4.61	128.0	16.0	64.0
Tc08	2.0	4.0	2.0	6.67 ± 2.65	2.0	3.33 ± 1.15	4.0	4.0
Tc10	26.67 ± 9.24	128.0	16.0	N/A	N/A	N/A	N/A	128.0
At01	64.0 ± 0.0	32.0 ± 0.0	16.0 ± 0.0	128.0 ± 0.0	16.0 ± 0.0	256.0 ± 0.0	16.0 ± 0.0	128.0 ± 0.0
At02	2.0 ± 0.0	5.33 ± 2.31	1.0 ± 0.0	3.33 ± 1.15	4.0 ± 0.0	1.0 ± 0.0	4.0 ± 0.0	1.0 ± 0.0
At03	16.0 ± 0.0	N/A	8.0 ± 0.0	N/A	N/A	32.0 ± 0.0	8.0 ± 0.0	32.0 ± 0.0
At04	N/A	N/A	256.0 ± 0.0	N/A	16.0 ± 0.0	N/A	N/A	N/A
Cs01	32.0	32.0	8.0	64.0	4.0	128.0	16.0	64.0
Zm01	13.33 ± 4.61	16.0	8.0	64.0	16	16.0	32.0	16.0
Cs02	8.0	N/A	3.33 ± 1.15	64.0	8.0	32.0	4.0	32.0
Cs03	128.0	N/A	64.0	N/A	128.0	16.0	128.0	N/A
Gr01	0.5	3.33 ± 1.15	1.0	1.0	0.83 ± 0.29	0.5	1.0	1.0
Gr02	4.0	32.0	1.0	4.0	1.0	1.0	2.0	4.0
Asc-8	2.0	4.0	1.0	4.0	2.0	3.33 ± 1.15	2.0	2.0
DS01	1.0	2.0	1.0	16.0	8.0	0.5	1.0	0.5
Ampicilin	10.77	N/A	0.08	N/A	5.38	1.35	2.69	1.35
Gentamicin	0.52	0.13	0.033	8.37	0.26	0.53	0.52	0.53

^*^Standard deviation equals 0 is not displayed in the table. N/A – No antimicrobial activity observed. Peptides Tc01, Tc04, Tc07, Tc09 and Tc11 did not show antimicrobial activity against any of the tested microorganisms.

The microbicidal activities of these IAPs were also evaluated by standard dilution assays and are presented as Supplementary Material (Supplementary Material 02, Tables S2 and S3). Inhibitory and microbicidal concentrations were very similar for the most active peptides, except for Tc06, At01, Cs01 and Zm01, which presented non-detectable microbicide concentrations to various microorganisms, while they inhibited their growth at concentrations lower than 32 µM, indicating a microbistatic activity rather than microbicidal.

The sixteen synthetic IAPs that displayed antimicrobial activity were tested for their cytotoxicity using human erythrocytes as model. Although the IAPs described herein were filtered to hinder the growth of plant pathogens (Table 1), erythrocyte lysis is used as a standard to evaluate peptide selectivity^16,17,26^. IAPs induced erythrocyte lysis at variable concentrations similar to the AMPs Asc-8^17^ and DS01^16^ (Table 6 and Supplementary Material 03, Table S4). While Asc-8 is a known hemolytic molecule, DS01 did not induce significant alterations in red or white blood cells at the tested concentrations up to 45 μM^16^. IAPs induced 50% hemolysis at intermediate concentrations to these two AMPs. Tc02, Gr01 and Gr02, three of the peptides with highest antimicrobial activities, induced 50% hemolysis at 64 μM, a concentration approximately 32 times higher than that required for fungicidal activity, or 12 times higher than that necessary for antibacterial activity.

**Table 6:** Hemolytic activity.

Peptides	Hemolytic activity of human red blood cells (%)
	SC50**
Tc02	64 µM
Tc03	>128 µM
Tc05	>128 µM
Tc06	>128 µM
Tc08	>128 µM
Tc10	>128 µM
At01	>128 µM
At02	>128 µM
At03	>128 µM
At04	>128 µM
Cs01	>128 µM
Zm01	>128 µM
Cs02	>128 µM
Cs03	>128 µM
Gr01	64 µM
Gr02	64 µM
Asc-8	16 µM
Ds01	>128 µM

^**^IAP concentration that at least 50% of red blood cells remain intact.

### IAPs from *T*. *cacao* inhibit the germination of *Moniliophthora perniciosa* basidiospores

**Figure 4:**
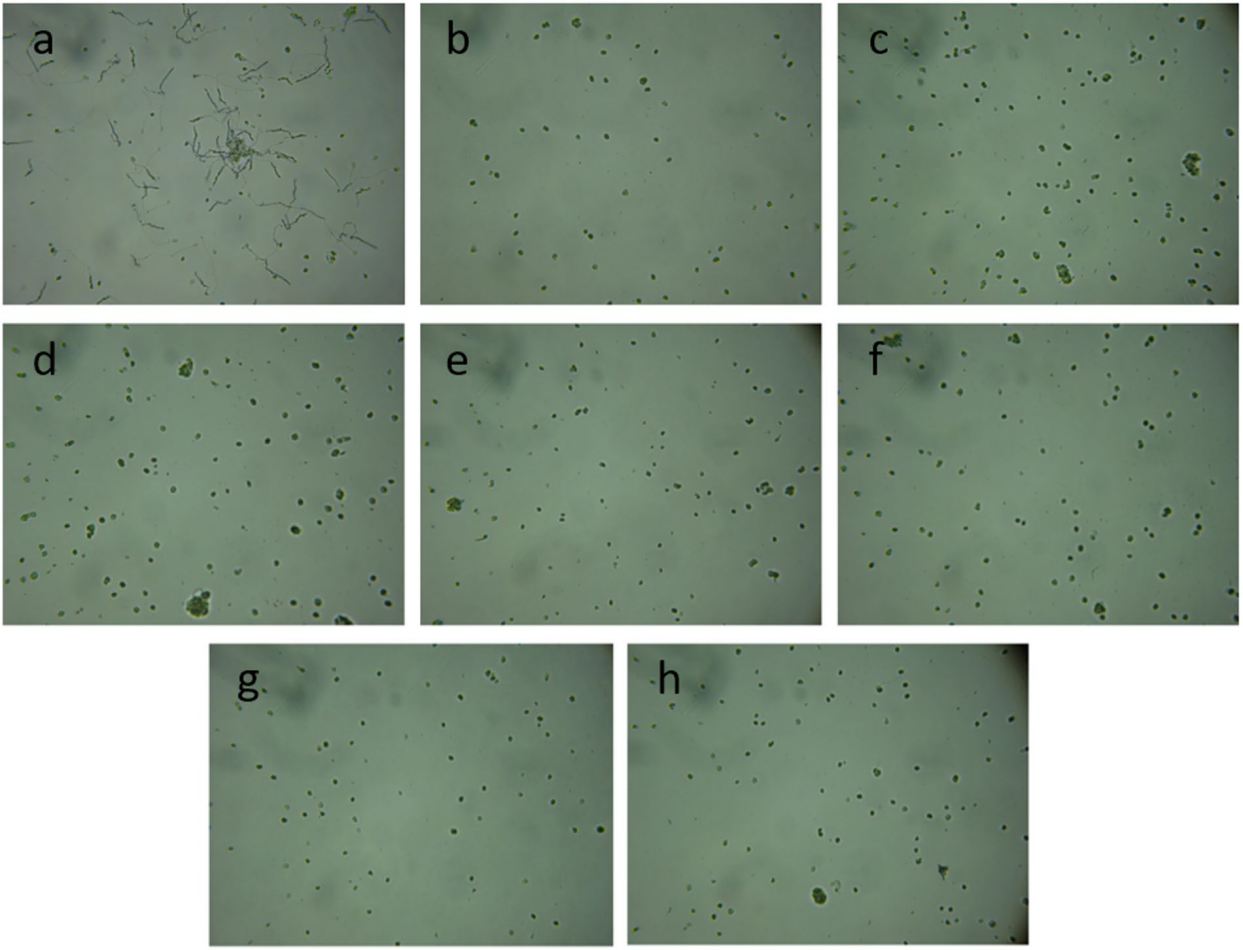
*Moniliophthora perniciosa* basidiospores germination test. After 4 hours of growth, the basidiospores were visualized. (**a**) Without peptides; (**b**) Tc02, 16 μM; (**c**) Tc05, 256 μM; (**d**) Tc06, 64 μM; (**e**). Tc08, 128 μM; (**f**) Tc10, 64 μM; (**g**). Asc-8, 16 μM; (**h**). DS01, 256 μM. The concentrations described for each peptide correspond to the minimum concentrations required to inhibit at least 95% basidiospore germination.

The eleven IAPs filtered from the *T*. *cacao* genome were evaluated for their potential to inhibit the germination of *M*. *perniciosa* basidiospores, the causal agent of witches’ broom disease^27^. *M*. *perniciosa* basidiospores germinate and infect cocoa meristems 4 hours after contact with cocoa meristems^28^. Five IAPs and the amphibian peptides DS01 and Asc-8 inhibited the germination of 1 × 10^6^ basidiospores after 4 hours of incubation in the evaluated concentrations, ranging from 256–0.5 µM (Fig. 4). No basidiospores germination were detected even with incubation for longer time periods (24 h and 48 h, Supplementary Material 04, Figure S2). Tc02, a fragment of the ARM repeat superfamily protein, putative isoform 1 from *T*. *cacao*, inhibited at least 95% basidiospore germination (IC_95_) of *M*. *perniciosa* basidiospores at 16 µM. Tc06 and Tc10 also inhibited significantly the growth of fungal spores, with an IC_95_ of 64 µM (Table 7).

**Table 7:** Inhibitory concentration of *T*. *cacao* IAPs on *M*. *perniciosa* basidiospores germination.

Peptides	Inhibitory concentration (µM)	
	IC95*	IC50**
Tc02	16	8
Tc05	256	128
Tc06	64	32
Tc08	128	64
Tc10	64	32
Asc-8	16	8
DS01	256	128

*Inhibitory concentration that at least 95% of basidiospores germination was inhibited. **Inhibitory concentration that at least 50% of basidiospores germination was inhibited.

### IAPs from *G*. *raimondii* inhibit areolate mildew development on cotton leaves

**Figure 5:**
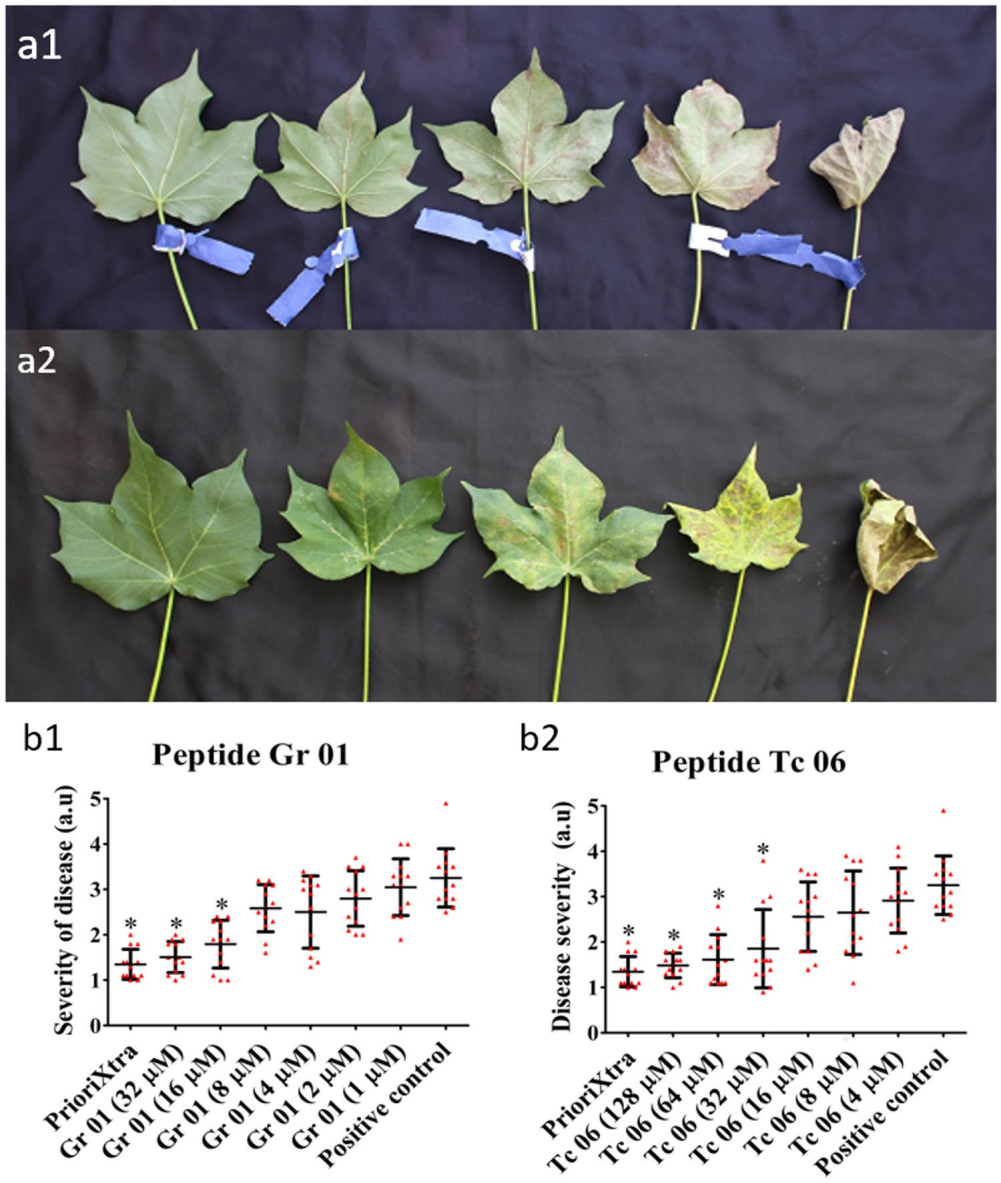
*Ramularia areola* infection test. Disease severity index ranging from 1 (without symptoms) and 5 (over 50% of leaf area showing symptoms) in both abaxial (**a1**) and adaxial surface (**a2**). Areolate mildew severity in cotton leaves after treatment with a commercial fungicide (PrioriXtra) and different concentrations of IAPs Gr01 (**b1**) and Tc06 (**b2**). Gr01 and Tc06 showed comparable results to fungicide at concentrations starting at 16 µM and 32 µM, respectively.

IAPs were also able to inhibit the cotton pathogen *Ramularia areola* ^29^ growth *in vitro* (Table 4). The IAPs Gr01 and Gr02, prospected from *G*. *raimondii* proteins, along with other peptides such as Tc06, At03 and Cs02, originally prospected for other plants but found with 100% identity in the cotton genome, were evaluated for their potential to inhibit disease development on cotton leaves infected with *R*. *areola* spores. Disease severity was evaluated in a scale from 1 (no symptoms) to 5 (over 50% of leaf area showing disease symptoms) (Fig. 5a). IAPs and 2 × 10^6^ spores were incubated for 20 minutes and this solution was applied in plant leaves. Gr01 and Tc06 showed significant results (p < 0.05, One-way ANOVA) on the control of areolate mildew down to 16 and 32 µM, respectively (Fig. 5b). Moreover, IAPs showed similar qualitative results to the commercial fungicide used as control. The number of spores collected from the leaves was also significantly decreased in the presence of the IAPs (Supplementary Material 05, Figure S3). Surprisingly, Gr02 did not show a significant reduction in areolate mildew severity and spores concentration. This demonstrates that *in vitro* biological activities might not be observed *in vivo*, and novel assays are necessary to accurately address these results.

## Discussion

We are persuaded that mature proteins represent a feasible and copious source from where smaller bioactive polypeptide chains may be earned, particularly antimicrobials. In the present report, we confirm our previous findings^7^ by demonstrating additional proofs of this concept exploring plant protein sequences as sources of their derived peptide primary structures and, evaluating their respective biological activities against varied pathogens. The IAPs searched in plant genomes described herein have equivalent potency, selectivity, and display a similar activity spectrum towards microorganisms as AMPs from various sources^15,30–32^. Moreover, these IAPs fold into α-helical segments upon membrane interaction producing a range of membrane disturbances, in the same manner as mature antimicrobial peptides from frog skin secretions^7,33^ (Figs 2 and 3). The disturbances introduced by the IAPs in model membranes, as depicted in Fig. 3, are typical of molecules that are localized at the polar/nonpolar interface of the phospholipid bilayer, with a tendency to disturb the acyl interior of membranes, similar to DS01 and other AMPs in the literature^34^. Furthermore, membrane disturbance is directly associated with peptide helicity, and the same is observed for the IAPs (Fig. 2). Our data demonstrates that classical antimicrobial peptides, selected for their membrane disruptive potential by millions of years of evolution^35^, can be mimicked in several aspects by internal protein segments with comparable physicochemical properties, but with no identifiable adaptive pressure for such activity when encrypted in parent proteins. Once identified and characterized, these IAPs can be used to confer resistance to a variety of pathogens, by transforming plants with fragments of their own genetic material, providing an alternative to the transgenic technology. This was demonstrated by our group^7^, where soybean IAPs were able to decrease symptons of Asian rust (*Phakopsora pachyrhizi*) on soybean leaves *in vitro* and *in vivo*.

To the best of our knowledge, this is the first time that molecules with significant antimicrobial activity are obtained from *T*. *cacao* and *G*. *raimondii*. The IAPs Tc02, Tc06 and Tc10 inhibited the germination of *M*. *perniciosa* basidiospores and can be considered promising agents against other *T*. *cacao* pathogens, such as *Phytophthora spp*. and *Moniliophthora roreri* ^36^. Despite the encouraging results, cocoa transformation is challenging due to its long growth period to yield its first fruits^37^. Micro-Tom tomatoes transformation represents an alternative to evaluate the intragenic approach in the control of *M*. *perniciosa* infection^38^. For cotton plants, the inhibition of areolate mildew symptoms (Fig. 5b1 and b2) and the decrease in spore concentration (Supplementary Material 05, Figure S3) with comparable results to a commercial fungicide indicates that peptides Gr01 and Tc06 are suitable candidates for cotton plant transformation.

The evidences we have encountered so far by applying our methodology, alongside most of the native bioactive peptide data available in the literature^7,16,31,39^, compel us to infer that other sources of proteins in animals, algae and microorganisms should yield similar results to the ones we have evaluated here. Putative IAPs can be prospected from both clade-specific proteins or from evolutionarily conserved homologous genes that span different taxa. The latter seems to be the case of the IAPs Tc06 and Cs02. Tc06 is derived from cytochrome P450 family 87, found with 100% identity in many eudicots and with more than 75% identity in some monocots such as *Oryza sativa* and *Zea mays* (Table 2), while the IAP Cs02 is a fragment of the photosystem II CP47 chlorophyll apoprotein, found identically in Bacteria and Eukarya kingdoms (Supplementary Material 06, Figure S4). However, at the present phase of our investigations it appears that more important than a most needed systematic search for novel bioactive peptides in other protein sources that would reinforce our findings, some fundamental questions seem to emerge from the current scenario that deserve to be addressed with higher priority: 1. Are these facts pointing out to a general phenomenon? 2. If so, what would be the biological and physiological implications of that? 3. Could protein degradation (into bioactive peptides) be considered an overlooked step of specific protein post-processing and/or function in nature and not only an earlier stage of proteolysis destined to supply amino acids to various metabolic pathways? 4. What would be the implications of this concept in biotechnology, agriculture and human health?

From our perspective, these questions have no easy answers. Each one of them appears to open such a wide range of theoretical and experimental research possibilities that, at present, are beyond our best work force and probably lifetime effort.

It is our understanding that although the empirical results on microbicidal activities presented here constitute a robust and assorted set of material evidences on which our prime hypothesis is anchored, they seem to represent just the tip of a much larger aspect of the reality that need to be investigated rigorously not only for different types of antimicrobial properties and modes of action, but also for other important biological functions such as analgesic, anti-inflammatory, hypotensive, for example. In fact, our group recently identified encrypted opioid peptides on dehydrin protein family from *Coffea spp*., showing similar results to Leu-Enkephalin^40,41^. We also identified a conserved Met-Enkephalin in a F-box protein from *T*. *cacao* (NCBI EOY06230.1 – Residues Lys 176 to Lys 184) with similar cleavage sites for serine proteases as found on human pro-enkephalins (unpublished data). Such results highlight how abundant encrypted peptides can be, suggest another facet to genome plasticity and therefore offer new extrinsic principles to biodiversity^42–44^. Mechanistic features and physicochemical studies on the antimicrobial peptides evaluated in this work are available as a second part of the current paper, entitled “Towards an experimental classification system for membrane active peptides”.

## Material and Methods

### IAPs search criteria and peptide synthesis

The predicted proteins from *Theobroma cacao* were downloaded from Cacao Genome Database (www.cacaogenomedb.org) on October 2013. *Arabidopsis thaliana*, *Gossypium raimondii* and *Citrus sinensis* genomes were downloaded from the Phytozome portal (https://phytozome.jgi.doe.gov) on February, July and August 2014, respectively. All genomes were searched for putative IAPs. IAPs were searched through a set of defined physicochemical parameters using Kamal alpha v.1.0 software^7^. Physicochemical descriptors were updated from our previous report and are listed in Table 1. A total of 21 peptides were chemically synthesized using Fmoc/t-butyl strategy^45^. Peptide chain elongation was performed on Rink Amide resin, yielding C-terminal amidated peptides. Two known AMPs from frog skin secretions, Dermaseptin DS01^16^ and Asc-8^17^, were also synthesized. Helical wheel plots were created using an Internet Tool (http://lbqp.unb.br/NetWheels/).

### Mass spectrometry analyses

All crude synthetic peptides were analyzed by mass spectrometry to confirm peptide mass and amino acid sequence. Experiments were carried out in an UltrafleXtreme MALDI-TOF/TOF (Bruker Daltonics), controlled by FlexControl 3.0 software (Bruker Daltonics) using α-cyano-4-hydroxycinnamic acid matrix (Fluka) for ionization. Peptides monoisotopic mass were obtained in reflector mode over a range of 700–3500 m/z with external calibration using Peptide Calibration Standard II (Bruker Daltonics). Peptide MS/MS spectra were obtained by means of LIFT fragmentation after analyzing the obtained MS spectra and selection of precursor ions for fragmentation. The software FlexAnalysis 3.0 (Bruker Daltonics) was used for mass spectrometric data analysis. Peptide primary structures were inferred by means of manual interpretation of fragmentation spectra.

### HPLC purification and peptide quantification

Reverse phase HPLC (RP-HPLC) of the synthetic peptides were performed in two different scales: analytical and preparative, both using C-18 columns, Grace Vydac 218TP54 and Grace Vydac 218TP1022, respectively. Ultrapure H_2_O + 0.1% (v/v) TFA (J.T. Baker) and acetonitrile acetonitrile (J.T. Baker) +0.1% (v/v) TFA (J.T. Baker) were used as solvent A and B, respectively. The chromatography method was set as a 50 minutes protocol composed of three steps: 1- 0–5 min: 5% (v/v) solvent B; 2- 5–45 min: gradient of solvent B up to 95% (v/v); 3- 45–50 min: 95% (v/v) of solvent B). Fractions were manually collected and analyzed by mass spectrometry to confirm the elution time of each synthetic peptide. Peptides were then purified in a preparative scale using the same parameters described above. Fractions of interest were collected and analyzed by mass spectrometry to confirm purity. Purified synthetic peptides containing Trp or, at least, three Tyr residues were quantified using calculated molar absorption coefficients^46^. The remaining peptides were quantified using the UV absorbance of the peptide bond according to the literature^47^.

### Large unilamelar vesicles (LUVs) preparation

DMPC and 2:1 DMPC:DMPG (w/w) were dissolved in chloroform and methanol (3:1 v/v) at 10 mg/mL, dried using a rotary evaporator and left 3 hours under high vacuum in a freeze dryer. Phospholipids were then dissolved in 20 mM Sodium phosphate – NaOH, 150 mM NaCl, pH 7.4 and hand-shaken until the formation of a cloudy solution, which was passed 19 times through a 100 nm polycarbonate membrane at 30 °C for the formation of large unilamelar vesicles (LUVs) using a mini-extruder (Avanti Polar Lipids). Phospholipid concentration was estimated according to the ammonium ferrothiocyanate method^48^.

### Differential scanning calorimetry (DSC)

Thermograms were obtained using a VP-DSC (GE Healthcare) at a temperature range from 10 to 40 °C at a scanning rate of 0.5 °C/min. Blank thermograms using buffer alone and 0.5 mM DMPC or 2:1 (w/w) DMPC:DMPG LUVs in buffer were acquired as reference. Peptides were added to fresh samples of 0.5 mM LUVs at a concentration of 20 μM (0.04 mol/mol peptide/phospholipids) at room temperature, immediately followed by DSC data acquisition. Each sample was subjected to various thermal scans until there were no distinguishable changes in the thermal profile of the main phase transition (P′_β_→L_α_) of phospholipids between scans. Data was concentration normalized, baseline subtracted (linear connect), and fitted to a non two-state transition with two peaks determined by the user applying the MicroCal Origin software v7.0. Re-scans for selected cases were acquired using fresh peptide and LUVs solutions to check the reproducibility of the data.

### Circular Dichroism (CD)

Experiments were conducted on a Jasco-J815 spectropolarimeter (Jasco International Co.). Spectra were acquired at room temperature from 200 to 260 nm as an average of 4 readings using a 0.1 cm path length cell, data pitch of 0.2 nm and a response time of 0.5 s. Data Scans of buffer and 2 mM DMPC and 2:1 DMPC:DMPG LUVs solutions were acquired and subtracted from each peptide data. Peptides were scanned at a concentration of 40 μM in buffer and then 50 fold excess of DMPC and 2:1 DMPC:DMPG LUVs were added, resulting in a molar ratio of 0.02 peptide/phospholipid. The spectra were converted to mean residue ellipticity and readings at [θ]_222_ nm were used to estimate helix percentages^49^.

### Evaluation of antimicrobial activity of IAPs

The susceptibility test of microorganisms was performed to evaluate the inhibitory activity of the IAPs. Protocols M7-A10^50^, M27-A3^51^ and M38-A2^52^ from Clinical & Laboratory Standards Institute (CLSI) were used for bacteria, yeasts and filamentous fungi tests, respectively. Briefly, different concentration of IAPs (256-0.5 µM) were tested against 16 microorganisms, in a final volume of 100 µL. Media conditions, final cell concentration and incubation conditions are presented in Supplementary Material 07, Table S5. Three biological repetitions, with 2 technical replicates each, were performed for each test, on polystyrene flat-bottom 96 wells microplates. The minimum inhibitory concentration (MIC) was defined as the concentration that no cells/hyphae were detected when visualized by optical microscopy.

The microbicidal activity of the IAPS was also evaluated. After the growing period showed on Supplementary Material 07 for each organism, 10 µL of the minimum inhibitory concentration and raising concentrations up to 256 µM were transferred to agar plates, and incubated at the same conditions (Table 2). YPD agar and Mueller-Hinton agar were used for fungi/yeasts and bacteria, respectively. Meanwhile, susceptibility microplates were incubated again to evaluate if there would be any growing after a longer period. For both approaches, the minimal killing concentration (MKC) was defined as the concentration that no growth was observed after incubation. Three biological replicates were performed for each test.

### Evaluation of cytotoxicity of IAPs

The cytotoxicity of the selected IAPS was performed using human red blood cells in strict accordance with relevant guidelines and regulations (Ethical committee – UnB # 1.939.989). Different concentrations of IAPs (128 µM–0.5 µM) were incubated with a final concentration of 2.5 × 10^5^ red blood cells.mL^−1^. Briefly, 100 µL of red blood cells was incubated with 100 µL of different concentrations of each IAP, prepared in PBS, for 30 minutes at 37 °C, with constant shaking at 120 rpm. PBS and Triton X-100 0.1% (v/v) were used as negative and positive controls, respectively. After incubation, the solution was centrifuged at 900 × g for five minutes followed by transferring 50 µL of the supernatant to flat-bottom polystyrene 96-well micro plates containing 50 µL of PBS. Microplate reading was performed at a BioTek Multireader, using a wavelength of 540 nm. All tests were performed in 3 biological replicates, composed of 3 biological repetitions each. The obtained data was subtracted by the negative control and relative to the positive control, defined as 100% lysis.

### Inhibition of *M*. *perniciosa* basidiospore germination by *T*. *cacao* IAPs

The antimicrobial potential of *T*. *cacao* IAPs for inhibiting *M*. *perniciosa* basidiospores was evaluated. Briefly, 1 × 10^6^ basidiospores mL^−1^ were incubated for one hour with different concentrations of *T*. *cacao* IAPs (256-0.5 µM), at 24 °C, in a final volume of 100 µL. Autoclaved dH_2_0 was used as a control. After incubation, 3 droplets of 10 µL each were placed in a slide coated with agar (1.2% (w/v)). Slides were incubated for 4, 24 and 48 hours at 24 °C. Slides were visualized using a DMRXA light microscope (Leica). Approximately 150–200 basidiospores per droplet from each time point were counted and compared to the control condition, which was defined as 100% germination. All experiments were performed in biological triplicates, with 3 technical repetitions each.

### IAP areolate mildew inhibition on cotton leaves

Different concentration of IAPs Tc06 (128-4 µM) and Gr01 (32-1 µM) were mixed with *Ramularia areola* spore solution (final concentration 2 × 10^6^ spores.mL^−1^) for 20 minutes prior to application on both surfaces of cotton leaves (cultivar IMA5675 B2RF). *R*. *areola* spores was also mixed with the commercial fungicide PrioriXtra (Syngenta®) (1,5 mL.L^−1^) as negative control for disease development, and with water, as positive control.

Experimental groups were composed of 13 pots, with two plants each. From each plant, two leaves from sixty one days old cotton plants were selected for treatment. Sixteen days after application, disease severity and number of spores per leaf per experimental group were evaluated. For severity analysis, a score from 1 (no symptoms) to 5 (symptoms over 50% of foliar area) was attributed for each leave. The final score for each treatment was an average from the four leaves from each pot. Spore counting was performed using a Neubauer chamber. Briefly, four leaves from each pot were scrapped and counted, yielding a final spore count per pot. Data and statistical analysis were performed using Excel, GraphdPad Prism and Past softwares.

## Electronic supplementary material

Supplementary Dataset 1
