# Supplementary material for: Encrypted Antimicrobial Peptides from Plant Proteins
Source: Sci Rep. 2017 Oct 16;7:13263. doi: 10.1038/s41598-017-13685-6 (PMC5643462; doi:10.1038/s41598-017-13685-6)
Supplement: Supplementary file 1 — Supplementary Dataset 1 [file 41598_2017_13685_MOESM1_ESM.pdf]

Encrypted Antimicrobial Peptides from Plant Proteins

Ramada, M.H.S.[1,2,3], Brand, G.D.[4], Abrão, F.Y.[5], Oliveira, M.[5], Cardozo Filho, J.L.[1,6], Galbieri, R.[6], Gramacho, K.P.[7], Prates, M.V.[1], Bloch Jr., C.[1*]

[1] Laboratório de Espectrometria de Massa, Embrapa Recursos Genéticos e Biotecnologia, 70770-917, Brasília, DF, Brazil.

[2] Departamento de Biologia Celular, Instituto de Ciências Biológicas, Universidade de Brasília, 70910-900, Brasília, DF, Brazil.

[3] Current Address: Pós-Graduação em Ciências Genômicas e Biotecnologia, Universidade Católica de Brasília, 70790-160, Brasília, DF, Brazil.

[4] Laboratório de Síntese e Análise de Biomoléculas, Instituto de Química, Universidade de Brasília, Brasília, DF, Brazil.

[5] Faculdade de Farmácia, FacUnicamps, 74535-280, Goiânia, GO, Brazil.

[6] Departamento de Fitopatologia, Instituto Mato-Grossense do Algodão, 78850-000, Primavera do Leste, MT, Brazil.

[7] Laboratório de Fitopatologia Molecular, Centro de Pesquisa do Cacau, 45600-970, Itabuna, BA, Brazil.

Supplementary Material

Supplementary Material 01, Table S1 and Figure S1

Table S1. Filtered IAPs physicochemical properties.

**Peptide**	**Amino acid sequence**	**Residues**	**Net charge**	**Molecular Mass**	**Isoeletric point**	**Hidrophobicity (Tm scale)**	**Hydrophobic moment (Tm scale)**	**Aggregation (Na4vSS)**	**Helix (GOR IV)**
Tc01	**VALRLAKEVIKVQQGW**	16	2	1836,09	10,54	-0,057	0,719	1,89	Não
Tc02	**GKILKYLLYLLRKYANLIIR**	20	5	2462,55	10,79	0,409	1,061	50,39	Sim
Tc03	**IKLRNVLKYLFRIDVIKEDIL**	21	2	2599,58	10,05	0,165	0,521	19,89	Sim
Tc04	**RVLKDVESALRESVANWKIVIG**	22	1	2480,41	9,21	-0,236	0,839	6,54	Sim
Tc05	**IVNHLVKLFDKGLNSIVNLR**	20	3	2290,34	10,54	0,123	1,018	15,97	Não
Tc06	**GSLHGFMYKYLKNMVLNLF**	19	3	2273,17	10,03	0,413	0,590	32,51	Não
Tc07	**LIKVVNHVQYNVTLHWHGIR**	20	5	2424,35	10,50	0,112	0,565	17,01	Não
Tc08	**LHRLVKLVAALLRGYASKVDTH**	22	5	2460,45	10,79	-0,055	0,875	14,14	Sim
Tc09	**GIVLKDLFSEKLRRYKIVIG**	20	3	2347,41	10,51	0,021	0,523	25,39	Sim
Tc10	**GLLFKELQKLIRYQIFIGK**	19	3	2305,39	10,52	0,313	0,677	35,32	Sim
Tc11	**LLDKLKRTLLSIEAVLI**	17	1	1936,23	9,21	0,302	0,883	20,99	Sim
At01	**GSLHGFMYKYLKNMVLTLF**	19	3	2260,17	10,31	0,015	0,015	38,53	Não
At02	**KVLSKVHTLLKAVLAL**	16	4	1731,13	10,84	0,001	0,195	37,60	Sim
At03	**GAKLAKKQVRALGKFFSF**	18	5	1994,18	11,85	0,118	0,281	5,95	Sim
At04	**GLYNFIKVLGRTVFGLYKQF**	20	3	2361,32	10,50	0,057	0,146	38,51	Não
Cs01	**GSLHGFMYRYLKNMVLNLF**	19	3	2301,17	10,19	-0,044	0,225	30,89	Não
Zm01	**GSLHGFMYKYLKTLVLRLY**	19	4	2300,27	10,32	0,046	0,270	39,45	Não
Cs02	**FFGHIWHGARTLFRDVFA**	18	3	2175,11	10,19	0,119	0,163	11,78	Sim
Cs03	**FFYNVIKIYGNMAGRISK**	18	3	2119,12	10,50	0,043	0,157	24,87	Não
Gr01	**GFKLGRKLVKVFKWII**	16	5	1930,22	11,85	0,001	0,234	36,74	Não
Gr02	**ANRLLEAYKMLLKFLGNLR**	19	3	2261,30	10,79	0,021	0,146	14,31	Sim
DS01	**GLWSTIKQKGKEAAIAAAKAAGQAALGAL**	29	3	2792,59	10,54	-0,264	0,252	-11,05	Sim
Asc-8	**GFKDLLKGAAKALVKTVLF**	19	3	2017,23	10,54	0,110	0,706	14,33	Sim

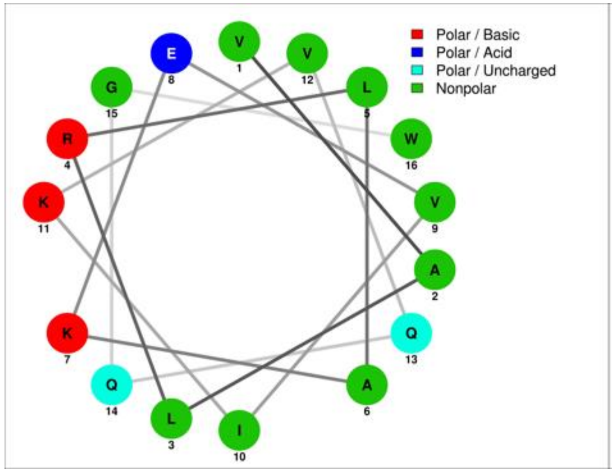

Tc 01

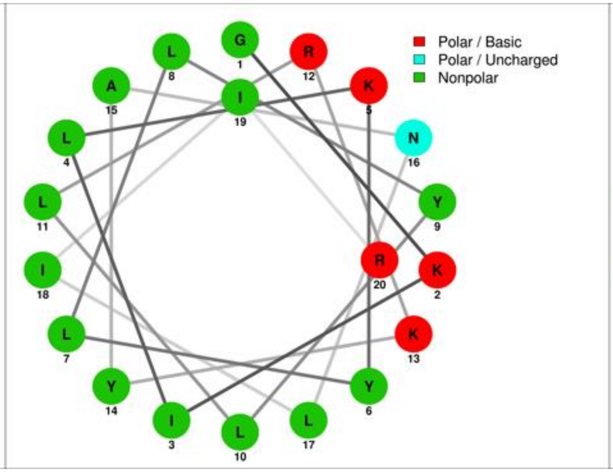

Tc02

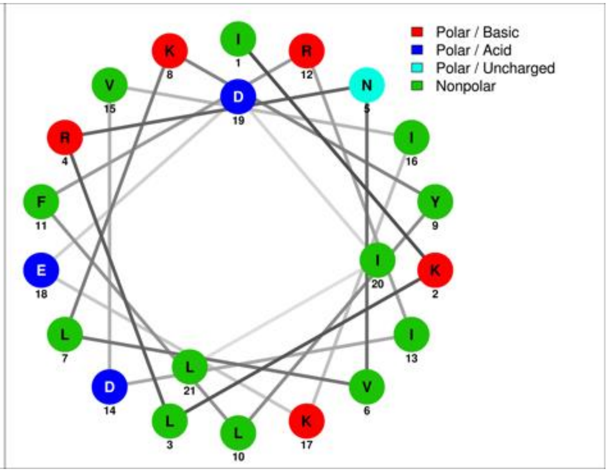

Tc03

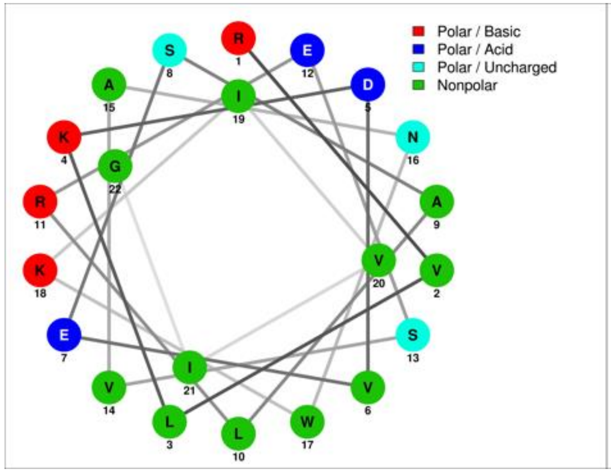

Tc04

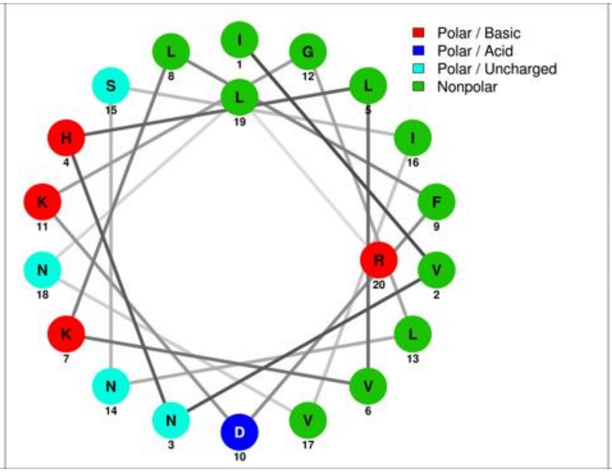

Tc05

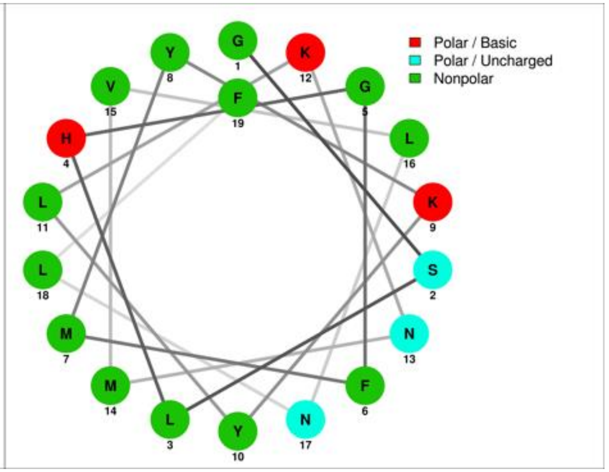

Tc06

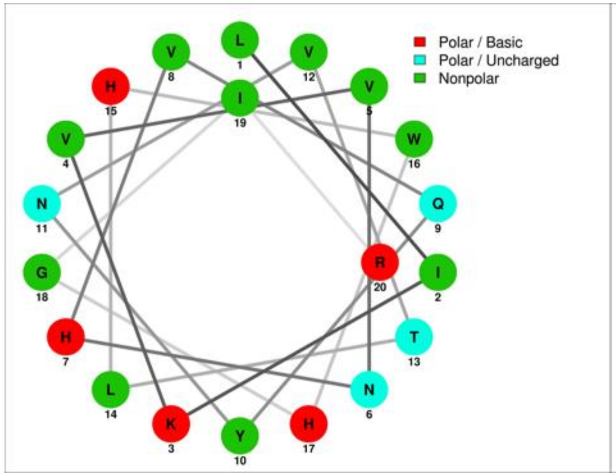

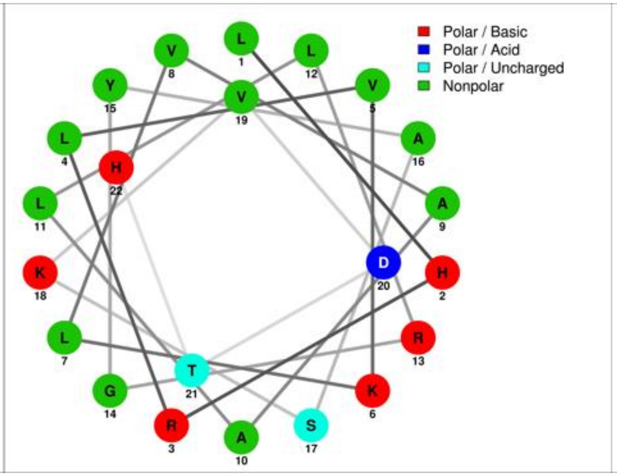

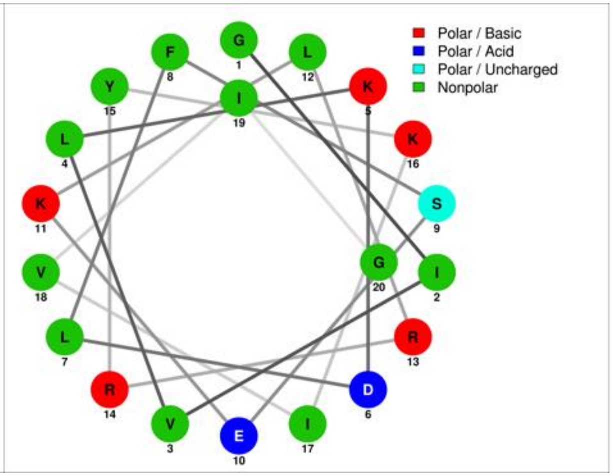

Tc 07 **Tc08** **Tc09**

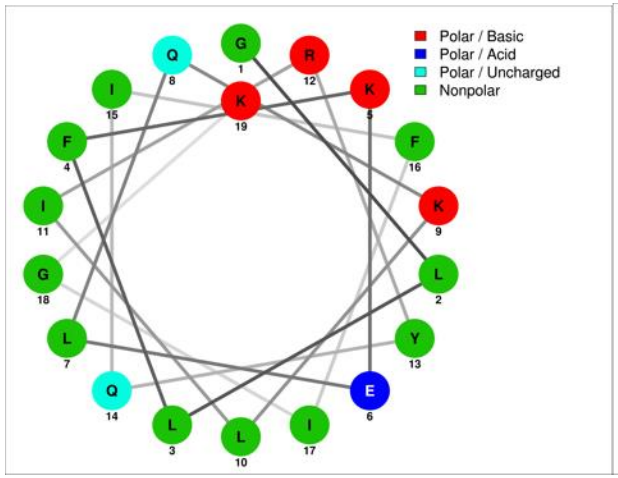

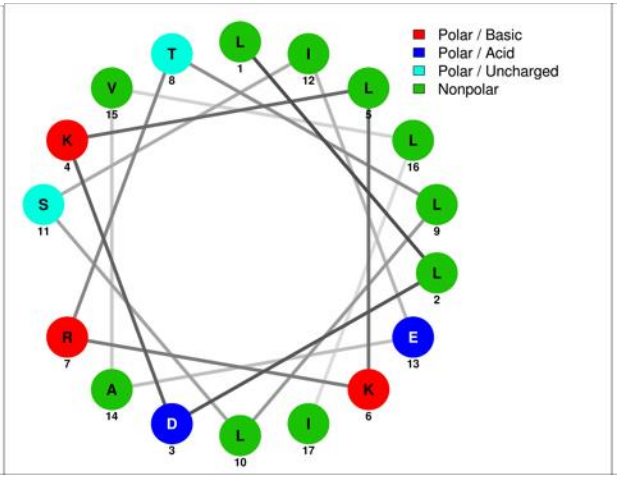

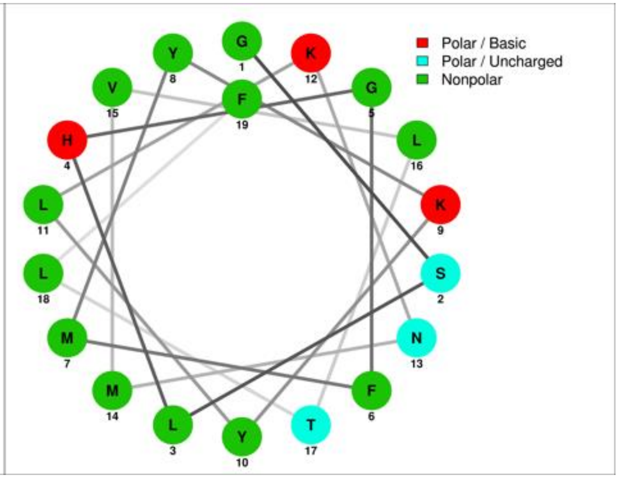

Tc10 **Tc11** **At01**

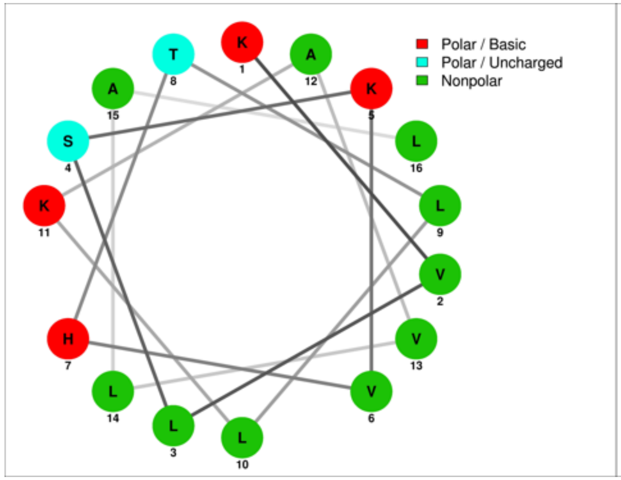

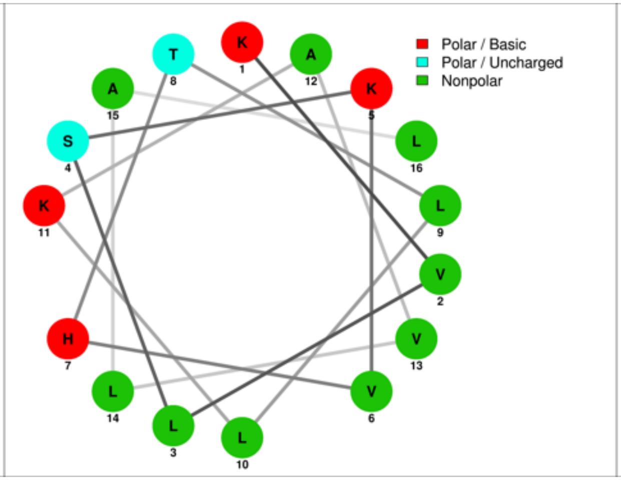

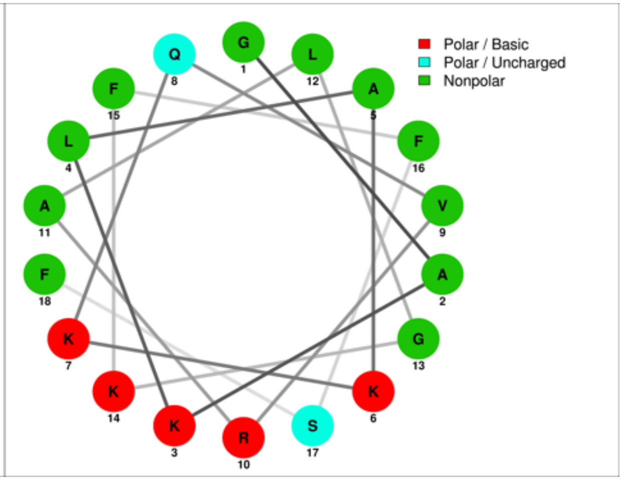

At 02

At03

At04

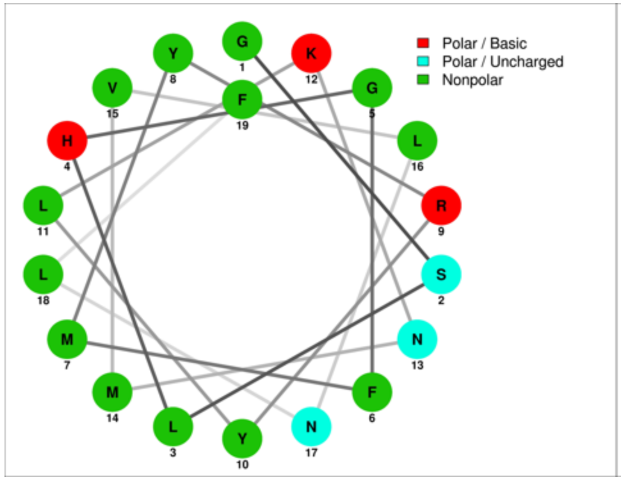

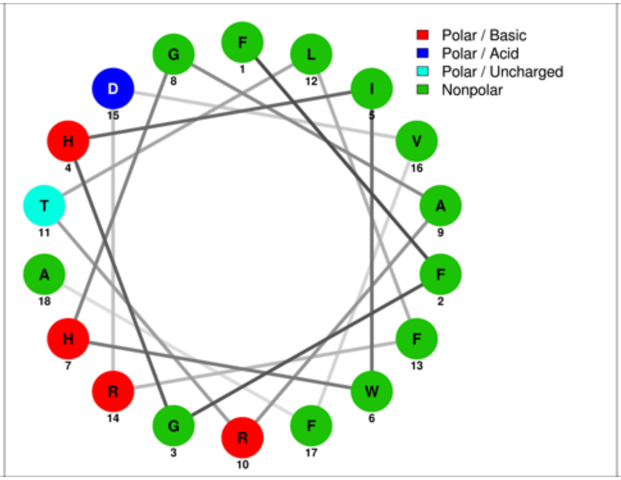

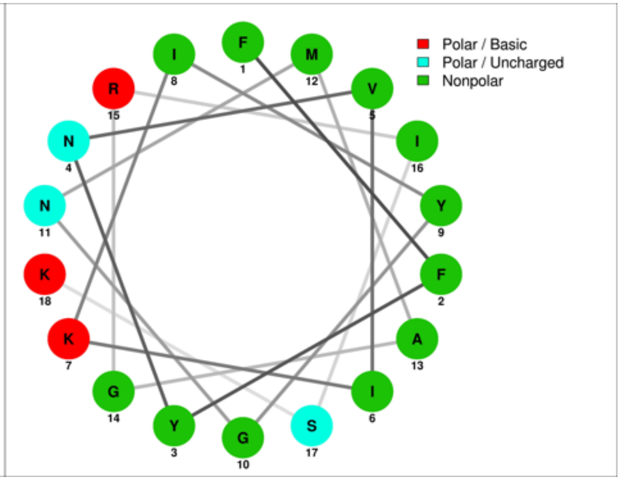

Cs01

Cs02

Cs03

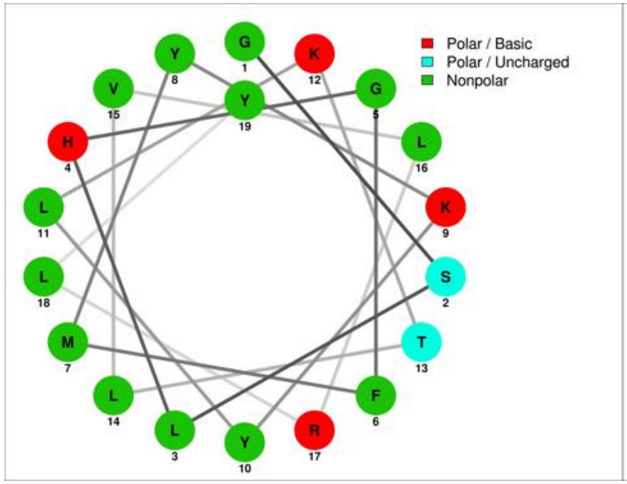

Zm01

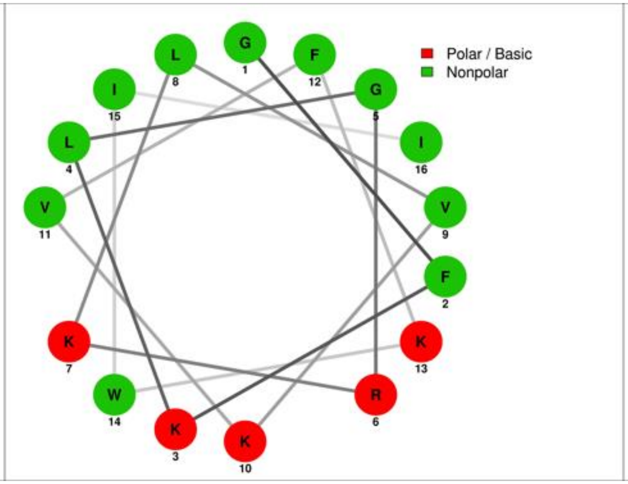

Gr01

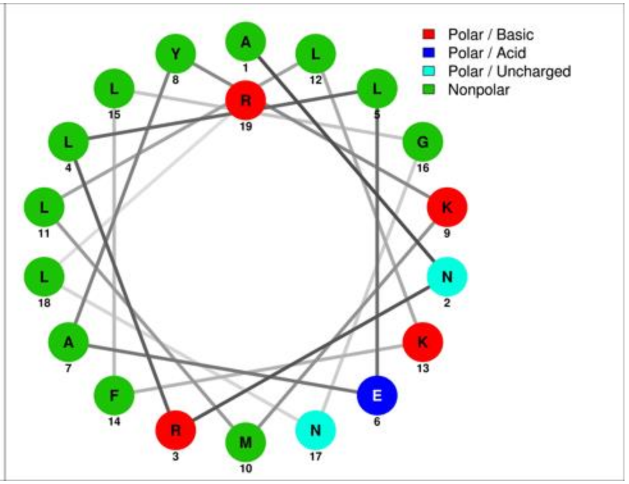

Gr02

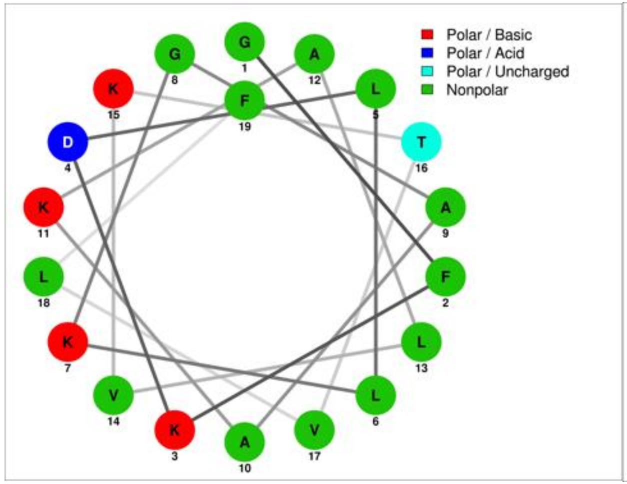

Asc-8

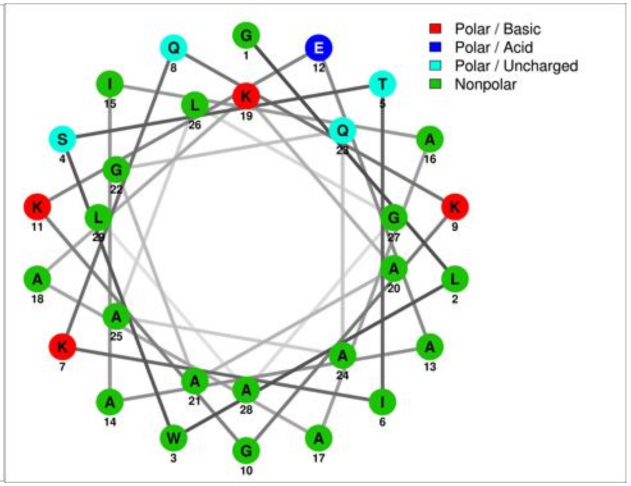

DS01

Figure S1. Helical wheel plot of the Intragenic Antimcrobial Peptides considered in this study.

Table S2. Fungicidal activity of IAPs against yeasts and filamentous fungi.

Peptides	Minimum microbicidal concentration (µM ± SD)							
	Candida albicans	*Cryptoccus neoformans*	*Fusarium solani*	*Fusarium oxysporum*	*Fusarium oxysporum vas*	*Ramularia areola*	*Rhizoctonia solani*	*Trichoderma asperellum*
Tc02	2.0 ± 0.0	1.0 ± 0.0	1.0 ± 0.0	2.0 ± 0.0	1.0 ± 0.0	0.83 ± 0.29	2.0 ± 0.0	2.0 ± 0.0
Tc03	N/A	N/A	N/A	N/A	N/A	N/A	N/A	N/A
Tc05	128.0 ± 0.0	256.0 ± 0.0	N/A	N/A	N/A	N/A	N/A	N/A
Tc06	4.0 ± 0.0	10.67 ± 4.61	N/A	N/A	64.0 ± 0.0	4.0 ± 0.0	N/A	N/A
Tc08	4.0 ± 0.0	8.0 ± 0.0	32.0 ± 0.0	64.0 ± 0.0	16.0 ± 0.0	8.0 ± 0.0	64.0 ± 0.0	32.0 ± 0.0
Tc10	128.0 ± 0.0	128.0 ± 0.0	N/A	N/A	N/A	128.0 ± 0.0	N/A	N/A
At01	26,67 ± 9.24	64 ± 0.0	N/A	N/A	128.0 ± 0.0	8.0 ± 0.0	N/A	N/A
At02	8.0 ± 0.0	4.0 ± 0.0	64.0 ± 0.0	32.0 ± 0.0	16.0 ± 0.0	4.0 ± 0.0	32.0 ± 0.0	16.0 ± 0.0
At03	128.0 ± 0.0	64.0 ± 0.0	N/A	N/A	256.0 ± 0.0	32.0 ± 0.0	N/A	N/A
At04	N/A	256.0 ± 0.0	N/A	N/A	N/A	N/A	N/A	N/A
Cs01	4.0 ± 0.0	8.0 ± 0.0	N/A	N/A	128.0 ± 0.0	4.0 ± 0.0	N/A	N/A
Zm01	6.67 ± 2.31	8.0 ± 0.0	128.0 ± 0.0	128.0 ± 0.0	64.0 ± 0.0	2.0 ± 0.0	N/A	N/A
Cs02	32.0 ± 0.0	32.0 ± 0.0	N/A	N/A	128.0 ± 0.0	32.0 ± 0.0	N/A	N/A
Cs03	128.0 ± 0.0	32.0 ± 0.0	N/A	N/A	N/A	64.0 ± 0.0	N/A	N/A
Gr01	1.0 ± 0.0	1.0 ± 0.0	1.0 ± 0.0	1.0 ± 0.0	1.0 ± 0.0	0.5 ± 0.0	2.0 ± 0.0	1.0 ± 0.0
Gr02	8.0 ± 0.0	5.33 ± 2.31	8.0 ± 0.0	8.0 ± 0.0	5.33 ± 2.31	2.0 ± 0.0	16.0 ± 0.0	8.0 ± 0.0
Asc-8	10.67 ± 4.61	4.0 ± 0.0	16.0 ± 0.0	16.0 ± 0.0	3.33 ± 1.15	4.0 ± 0.0	32.0 ± 0.0	4.0 ± 0.0
DS01	8.0 ± 0.0	8.0 ± 0.0	64.0 ± 0.0	64.0 ± 0.0	16.0 ± 0.0	8.0 ± 0.0	128.0 ± 0.0	8.0 ± 0.0
Fluconazole	NA	1.63 ± 0.0	N/A	N/A	N/A	N/A	N/A	N/A
Amphotericin B	0.54 ± 0.0	0.54 ± 0.0	1.08 ± 0.0	1.08 ± 0.0	1.08 ± 0.0	0.54 ± 0.0	2.16 ± 0.0	2.16 ± 0.0

Table S3. Bactericidal activity of IAPs against Gram-positive and negative bacteria.

Peptides	Minimum microbicidal concentration (μM ± SD)							
	Escherichia coli	*Pseudomonas aeruginosa*	*Bacillus subtilis*	*Bacillus cereus*	*Staphylococcus aureus*	Erwinia carotovora	*Pseudomonas syringae pv. tabaci*	*Xanthomonas campestris*
Tc02	10.67 ± 4.61	32.0 ± 0.0	16.0 ± 0.0	32.0 ± 0.0	10.67 ± 4.61	32.0 ± 0.0	8.0 ± 0.0	16.0 ± 0.0
Tc03	128.0 ± 0.0	N/A	16.0 ± 0.0	256.0 ± 0.0	N/A	64.0 ± 0.0	N/A	256.0 ± 0.0
Tc05	N/A	N/A	64.0 ± 0.0	N/A	N/A	N/A	N/A	N/A
Tc06	64.0 ± 0.0	N/A	64.0 ± 0.0	128.0 ± 0.0	26.67 ± 9.24	128.0 ± 0.0	128.0 ± 0.0	N/A
Tc08	6.67 ± 2.65	8.0 ± 0.0	8.0 ± 0.0	16.0 ± 0.0	6.67 ± 2.65	4.0 ± 0.0	16.0 ± 0.0	8.0 ± 0.0
Tc10	64.0 ± 0.0	N/A	16 ± 0.0	N/A	N/A	N/A	N/A	128.0 ± 0.0
At01	N/A	N/A	64.0 ± 0.0	256.0 ± 0.0	128.0 ± 0.0	128.0 ± 0.0	128.0 ± 0.0	N/A
At02	3.33 ± 1.15	8.0 ± 0.0	4.0 ± 0.0	8.0 ± 0.0	4.0 ± 0.0	4.0 ± 0.0	8.0 ± 0.0	6.67 ± 2.31
At03	32.0 ± 0.0	N/A	8.0 ± 0.0	N/A	N/A	32.0 ± 0.0	N/A	64.0 ± 0.0
At04	N/A	N/A	256.0 ± 0.0	N/A	N/A	N/A	N/A	N/A
Cs01	42.67 ± 18.47	N/A	8.0 ± 0.0	64.0 ± 0.0	5.53 ± 2.31	128.0 ± 0.0	128.0 ± 0.0	N/A
Zm01	16 ± 0.0	32.0 ± 0.0	8.0 ± 0.0	64.0 ± 0.0	16 ± 0.0	32.0 ± 0.0	64.0 ± 0.0	256.0 ± 0.0
Cs02	16 ± 0.0	N/A	4.0 ± 0.0	128.0 ± 0.0	16 ± 0.0	64.0 ± 0.0	128.0 ± 0.0	64.0 ± 0.0
Cs03	128.0 ± 0.0	N/A	128.0 ± 0.0	N/A	256.0 ± 0.0	32.0 ± 0.0	256.0 ± 0.0	N/A
Gr01	0.67 ± 0.29	3.33 ± 1.15	1.0 ± 0.0	4.0 ± 0.0	0.83 ± 0.29	2.0 ± 0.0	2.0 ± 0.0	1.0 ± 0.0
Gr02	8.0 ± 0.0	32.0 ± 0.0	2.0 ± 0.0	8.0 ± 0.0	8.0 ± 0.0	4.0 ± 0.0	3.33 ± 1.15	3.33 ± 1.15
Asc-8	2.0 ± 0.0	4.0 ± 0.0	2.0 ± 0.0	4.0 ± 0.0	2.0 ± 0.0	2.0 ± 0.0	4.0 ± 0.0	1.0 ± 0.0
DS01	1.0 ± 0.0	2.0 ± 0.0	1.0 ± 0.0	16.0 ± 0.0	8.0 ± 0.0	0.5 ± 0.0	4.0 ± 0.0	0.5 ± 0.0
Ampicilin	10.77 ± 0.0	N/A	0.08 ± 0.0	N/A	10.77 ± 0.0	2.70 ± 0.0	10.77 ± 0.0	5.38 ± 0.0
Gentamicin	0.52 ± 0.0	0.52 ± 0.0	0.033 ± 0.0	8.37 ± 0.0	0.52 ± 0.0	0.53 ± 0.0	0.52 ± 0.0	0.53 ± 0.0

Table S4. Hemolytic activity of IAPs on human red blood cells.

Peptides	Hemolytic activity (% ± SD)*							
	128 μM	64 μM	32 μM	16 μM	8 μM	4 μM	2 μM	1 μM
Tc02	50.15 ± 5.15	29.15 ± 2.57	17.39 ± 1.15	6.30 ± 1.59	2.89 ± 1.12	0.40 ± 0.26	0.00 ± 0.00	0.00 ± 0.00
Tc03	7.89 ± 1.44	2.29 ± 1.18	0.67 ± 0.30	0.09 ± 0.01	0.17 ± 0.04	0.00 ± 0.00	0.04 ± 0.03	0.03 ± 0.00
Tc05	3.37 ± 0.38	0.78 ± 0.47	0.21 ± 0.09	0.50 ± 0.10	0.07 ± 0.04	0.15 ± 0.05	0.07 ± 0.01	0.11 ± 0.02
Tc06	7.67 ± 1.69	1.18 ± 0.23	0.77 ± 0.23	0.01 ± 0.00	0.00 ± 0.00	0.00 ± 0.00	0.00 ± 0.00	0.00 ± 0.00
Tc08	44.15 ± 5.64	28.81 ± 2.67	15.47 ± 1.58	2.00 ± 1.13	0.30 ± 0.05	0.27 ± 0.10	0.11 ± 0.09	0.11 ± 0.04
Tc10	4.56 ± 0.58	0.68 ± 0.17	0.23 ± 0.10	0.00 ± 0.00	0.06 ± 0.02	0.18 ± 0.04	0.00 ± 0.0	0.00 ± 0.0
At01	14.6 ± 2.14	5.04 ± 2.39	1.05 ± 0.22	0.19 ± 0.01	0.00 ± 0.00	0.00 ± 0.00	0.00 ± 0.00	0.00 ± 0.00
At02	23.10 ± 3.41	10.28 ± 4.3	2.41 ± 0.98	0.30 ± 0.16	0.00 ± 0.00	0.02 ± 0.00	0.03 ± 0.00	0.04 ± 0.00
At03	0.34 ± 0.11	0.17 ± 0.08	0.03 ± 0.00	0.00 ± 0.00	0.02 ± 0.00	0.04 ± 0.01	0.00 ± 0.00	0.00 ± 0.00
At04	13.8 ± 2.41	4.12 ± 1.41	0.91 ± 0.20	0.40 ± 0.10	0.09 ± 0.04	0.00 ± 0.00	0.00 ± 0.00	0.00 ± 0.00
Cs01	8.01 ± 2.16	2.18 ± 1.62	0.31 ± 0.15	0.27 ± 0.09	0.09 ± 0.01	0.01 ± 0.00	0.05 ± 0.03	0.10 ± 0.04
Zm01	15.72 ± 3.48	6.56 ± 1.87	2.45 ± 1.03	0.66 ± 0.14	0.00 ± 0.00	0.00 ± 0.00	0.02 ± 0.00	0.00 ± 0.00
Cs02	3.41 ± 0.84	1.27 ± 0.44	0.00 ± 0.00	0.00 ± 0.00	0.00 ± 0.00	0.00 ± 0.00	0.00 ± 0.00	0.00 ± 0.00
Cs03	14.51 ± 1.55	4.11 ± 1.22	0.99 ± 0.34	0.21 ± 0.05	0.00 ± 0.00	0.01 ± 0.00	0.02 ± 0.00	0.00 ± 0.00
Gr01	51.72 ± 7.33	33.99 ± 5.46	11.33 ± 1.66	3.82 ± 1.28	1.96 ± 0.89	0.30 ± 0.15	0.00 ± 0.00	0.09 ± 0.01
Gr02	68.42 ± 5.45	46.44 ± 7.63	28.33 ± 4.77	7.01 ± 3.48	4.19 ± 0.78	1.23 ± 0.05	0.38 ± 0.09	0.01 ± 0.01
Asc-8	101.56 ± 9.52	91.90 ± 6.36	54.35 ± 4.5	28.10 ± 4.76	14.72 ± 0.79	0.26 ± 0.2	0.32 ± 0.1	0.00 ± 0.00
Ds01	14.61 ± 1.20	3.70 ± 1.34	0.79 ± 0.30	0.48 ± 0.12	0.03 ± 0.01	0.17 ± 0.04	0.19 ± 0.02	0.00 ± 0.00

* Experimental data expressed relative to positive control (Triton X-100 0.1%, w/v).

Supplementary Material 04, Figure S2

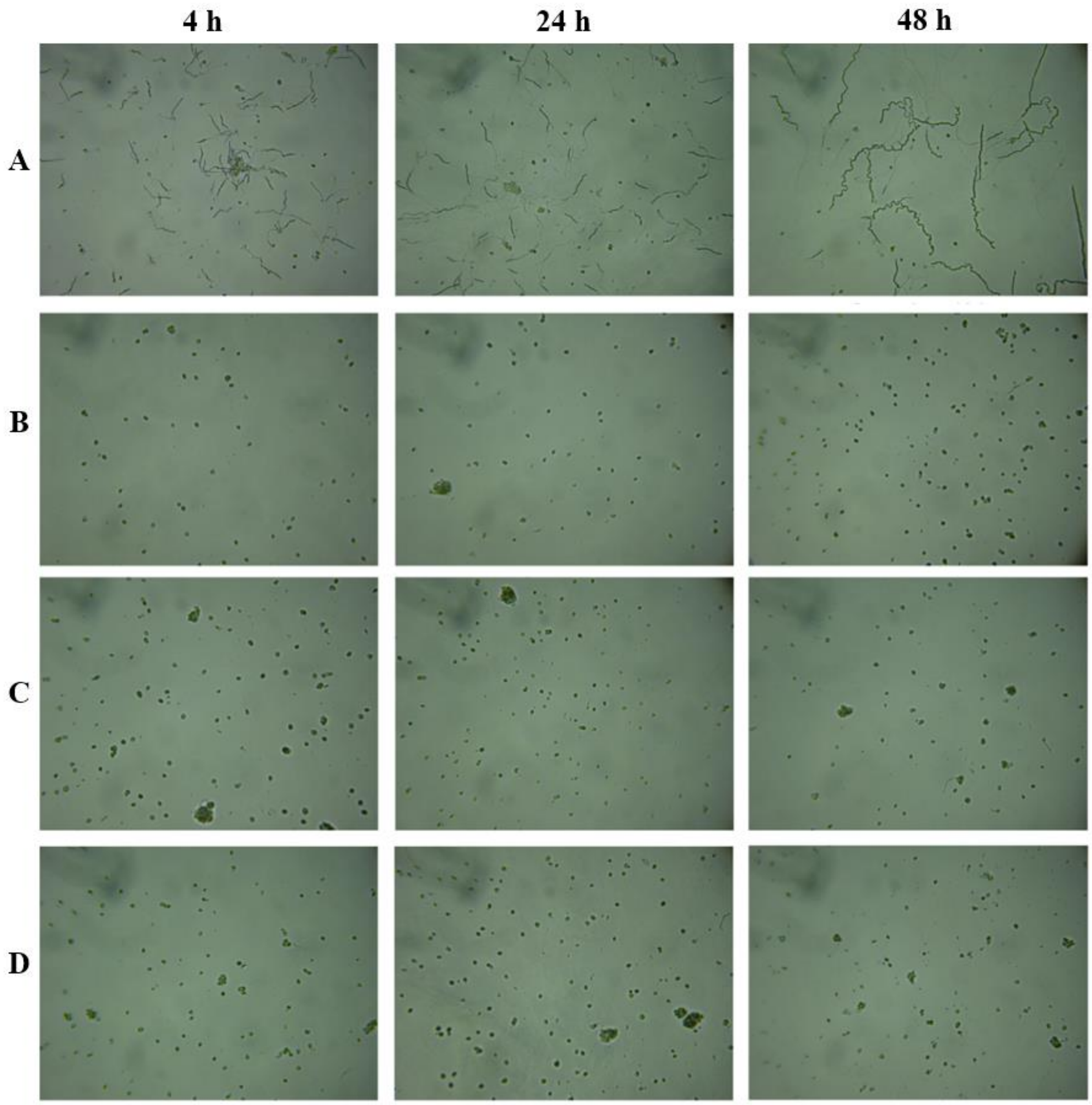

Figure S2. Timeline of *Moniliophthora perniciosa* basidiospores germination test. The most active peptides (B, C and D) show their potential to inhibit the basidiospores germination after 48 hours when compared to the control condition (A). A – without IAP; B- Tc02 (16 µM); C- Tc06 (64 µM); D- Tc10 (64 µM).

Supplementary Material 05, Figure S3

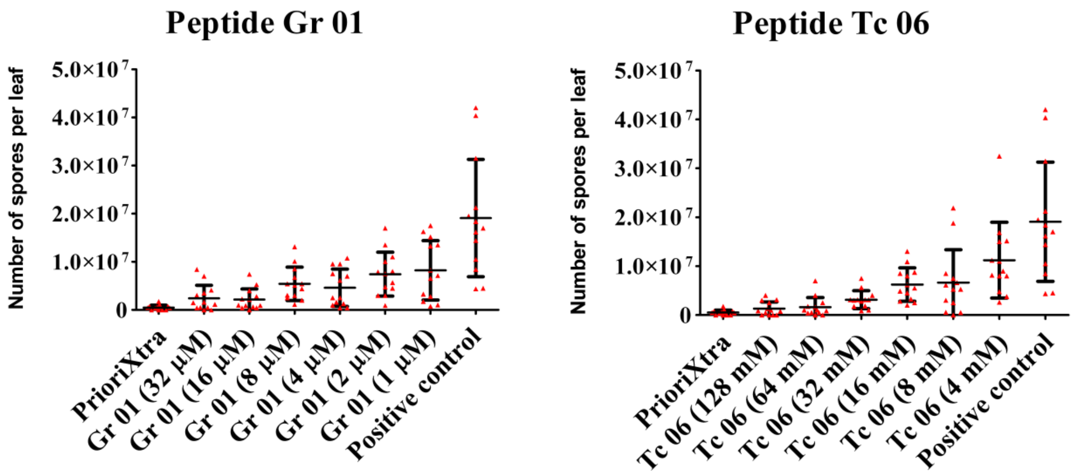

Figure S3. Number of spores of *Ramularia areola* per leaf after treatment with a commercial fungicide (PrioriXtra, 1.5mL.mL^{-1}) or different concentrations of IAPs Gr01 and Tc06.

Supplementary Material 06, Figure S4

Organism	NCBI Number	Aligned Sequence	N.r.[a]
C. sinensis	YP_740502.1	PRGWFTFGH-ASF-ALLF**FFGHIWHGARTLFRDVFA**	480
T. cacao	YP_004021342.1	PRGWFTFGH-ASF-ALLF**FFGHIWHGARTLFRDVFA**	480
G. raimondii	YP_005087719.1	PRGWFTFGH-ASF-ALLF**FFGHIWHGARTLFRDVFA**	480
A. thaliana	NP_051084.1	PRGWFTFGH-ASF-ALLF**FFGHIWHGARTLFRDVFA**	480
P. hollandica[b]	WP_026099548.1	TRGWFTFGH-ACF-ALLF**FFGHIWHGARTLFRDVFA**	488
Prasynophiceae sp.	AID67774.1	PRGWFTFGHLQ-F-ALLF**FFGHIWHGARTLFRDVFA**	480
O. elata	AAC90628.1	PRGWFTFGH-ASFALLFF**FFGHIWHGARTLFRDVFA**	45
C. falcatum	YP_009192055.1	PRGWFTFGH-GTFALIF-**FFGHIWHGARTLFRDVFA**	480
P. aquilinum	YP_003795606.1	PRGWFTFGH-ATFALIF-**FFGHIWHGARTLFRDVFA**	480
L. japonicum	YP_008474500.1	PRGWFTFGH-ATFALIF-**FFGHIWHGARTLFRDVFA**	480
P. patens	NP_904175.1	PRGWFTFGH-ATFALLF-**FFGHIWHGARTLFRDVFA**	480
A. officinalis	XP_020271588.1	PRGWFTFGH-ATFALLF-**FFGHIWHGARTLFRDVFA**	294
C. arietinum	YP_002149758.1	PRGWFTFGH-VSFALLF-**FFGHIWHGARTLFRDVFA**	480
C. cajan	YP_009309181.1	PRGWFTFGH-ASFALLF-**FFGHIWHGARTLFRDVFA**	485
G. stenophita	YP_008145817.1	PRGWFTFGH-ASFALLF-**FFGHIWHGARTLFRDVFA**	480
G. falcata	YP_008146063.1	PRGWFTFGH-ASFALLF-**FFGHIWHGARTLFRDVFA**	480
G. syndetika	YP_008146145.1	PRGWFTFGH-ASFALLF-**FFGHIWHGARTLFRDVFA**	480
G. max	YP_538791.1	PRGWFTFGH-ASFALLF-**FFGHIWHGARTLFRDVFA**	480
G. aurea	EPS74501.1	PRGWFTFGH-ASFALLF-**FFGHIWHGARTLFRDVFA**	480
U. gibba	YP_008082607.1	PRGWFTFGH-ASFALLF-**FFGHIWHGARTLFRDVFA**	480
A. duranensis	XP_015940753.1	PRGWFTFGH-ASFALLF-**FFGHIWHGARTLFRDVFA**	398
B. bealei	YP_008592666.1	PRGWFTFGH-ASFALLF-**FFGHIWHGARTLFRDVFA**	480
Z. violacea	ABC66113.1	PRGWFTFGH-ASFALLF-**FFGHIWHGARTLFRDVFA**	52
F. racemosa	YP_009176029.1	PRGWFTFGH-ASFALLF-**FFGHIWHGARTLFRDVFA**	488
E.minuta	AGW04541.1	PRGWFTFGH-ASFALLF-**FFGHIWHGARTLFRDVFA**	480
		******************	

[a] Number of last aminoacid residue shown of the whole protein sequence
[b] Bacteria

Figura S4. Cs02 IAP peptide alignment highlights conservation in different plants and Bacteria. This peptide is widely conserved in Bacteria and Eukarya.

Supplementary Material 07, Table S5

Table S5. Microorganisms and growth conditions.

Microorganisms	Incubation Temperature (°C)	Incubation Time (h)	Starting cell concentration	Growth Medium
Candida albicans ATCC 90028	37	48	2.5×10^3	RPMI 1640 pH 7.0
Cryptococcus neoformans ATCC 28957	37	72	2.5×10^3	RPMI 1640 pH 7.0
Escherichia coli ATCC 25922	37	24	5.0×10^5	Mueller-Hinton
Staphylococcus aureus ATCC 25923	37	24	5.0×10^5	Mueller-Hinton
Bacillus cereus ATCC 14579	37	24	5.0×10^5	Mueller-Hinton
Bacillus subtilis ATCC 23857	37	24	5.0×10^5	Mueller-Hinton
Pseudomonas aeruginosa ATCC 27853	37	24	5.0×10^5	Mueller-Hinton
Erwinia carotovora	28	24	5.0×10^5	Mueller-Hinton
Xanthomonas campestris pv. *citri*	28	24	5.0×10^5	Mueller-Hinton
Pseudomonas syringae pv. *tabaci*	28	24	5.0×10^5	Mueller-Hinton
Fusarium oxysporum	28	48	2.5×10^3	RPMI 1640 pH 7.0
Fusarium oxysporum f. sp. *vasinfectum*	28	72	2.5×10^3	RPMI 1640 pH 7.0
Fusarium solani	28	48	2.5×10^3	RPMI 1640 pH 7.0
Rhizoctonia solani	28	48	2.5×10^3	RPMI 1640 pH 7.0
Ramularia areola	25	120	2.5×10^3	RPMI 1640 pH 7.0
Trichoderma asperellum	28	48	2.5×10^3	RPMI 1640 pH 7.0
